# RegCloser: a robust regression approach to closing genome gaps

**DOI:** 10.1186/s12859-023-05367-0

**Published:** 2023-06-13

**Authors:** Shenghao Cao, Mengtian Li, Lei M. Li

**Affiliations:** 1 National Center of Mathematics and Interdisciplinary Sciences, Academy of Mathematics and Systems Science, Chinese Academy of Sciences, Beijing, 100190 China; 2 University of Chinese Academy of Sciences, Beijing, 100049 China

**Keywords:** Genome assembly, Closing gaps, Robust regression, Tandem repeat

## Abstract

**Background:**

Closing gaps in draft genomes leads to more complete and continuous genome assemblies. The ubiquitous genomic repeats are challenges to the existing gap-closing methods, based on either the k-mer representation by the de Bruijn graph or the overlap-layout-consensus paradigm. Besides, chimeric reads will cause erroneous k-mers in the former and false overlaps of reads in the latter.

**Results:**

We propose a novel local assembly approach to gap closing, called RegCloser. It represents read coordinates and their overlaps respectively by parameters and observations in a linear regression model. The optimal overlap is searched only in the restricted range consistent with insert sizes. Under this linear regression framework, the local DNA assembly becomes a robust parameter estimation problem. We solved the problem by a customized robust regression procedure that resists the influence of false overlaps by optimizing a convex global Huber loss function. The global optimum is obtained by iteratively solving the sparse system of linear equations. On both simulated and real datasets, RegCloser outperformed other popular methods in accurately resolving the copy number of tandem repeats, and achieved superior completeness and contiguity. Applying RegCloser to a plateau zokor draft genome that had been improved by long reads further increased contig N50 to 3-fold long. We also tested the robust regression approach on layout generation of long reads.

**Conclusions:**

RegCloser is a competitive gap-closing tool. The software is available at https://github.com/csh3/RegCloser. The robust regression approach has a prospect to be incorporated into the layout module of long read assemblers.

**Supplementary Information:**

The online version contains supplementary material available at 10.1186/s12859-023-05367-0.

## Background

Closing gaps in draft genomes, as an important step in the de novo assembly pipeline, remains a challenge due to the ubiquitous repetitive elements in genomes. Repetitive elements, as well as chimeric reads, cause ambiguities such as false alignments that disrupt the local assembly in gaps. Particularly, it is very difficult to resolve the copy number of a tandem repeat whenever it occurs [1]. Third Generation Sequencing (TGS) technology is expected to resolve the repeat problem if its long reads can span the repeat regions. Nevertheless, many genomes have been or are still sequenced, partially or fully, by Second Generation Sequencing (SGS) technology [2–5], due to its high accuracy, low cost, and wide availability. It is desirable to reconstruct, at least some, regions of repetitive elements by the SGS reads. Notably, the insert size of paired-end or mate-pair reads carries genomic information that can be utilized to facilitate assembly.

Most existing gap-closing methods including Sealer [6], GapCloser [7], and GapFiller [8] are based on the de Bruijn graph (DBG) approach. Although DBG-based methods are computationally efficient, they cut reads into k-mers so that some genomic information in raw reads are lost. In contrast, the overlap-layout-consensus (OLC) approach such as Phrap [9] directly aligns the original reads to detect overlaps. But finding a Hamiltonian cycle in the overlap graph is generally NP-complete [10], and heuristic algorithms are error-prone to false overlaps caused by either repeats or chimeric reads.

In this paper, we propose a new local assembly approach, referred to as RegCloser, to closing gaps. When it searches overlaps between reads from a gap region, insert-size information is utilized to guide the pairwise alignment to reduce the ambiguities caused by repeats as well as the time complexities. Then, RegCloser adopts a linear regression model to represent the detected overlaps. The motivation is to assign a coordinate axis to the DNA sequence due to its natural linear structure. Thus, read positions on the genome are represented as coordinates to be estimated and overlaps are represented as observations on the read coordinates’ differences.

Under the linear regression framework, the local DNA assembly is formalized as a parameter estimation problem. The estimation can be solved by well-established robust statistical methods [11]. In RegCloser, we customize a two-step robust regression procedure. In Step 1, it finds the read locations and detects false overlaps simultaneously by computing a robust M-estimate, which minimizes the Huber loss function. The loss function is convex so that its global optimum can be achieved by an iterative algorithm. Each iteration is to solve a sparse system of linear equations. In the M-estimation, the influence function of any outlier is bounded. In Step 2, RegCloser eliminates the influences of the false overlaps by a trimmed least squares estimate.

We will illustrate how RegCloser resolves tandem repeats by a real data example. Tests on simulated and real sequence data show that RegCloser outperforms several other popular gap-closing methods, especially in the presence of tandem repeats. Its applicability to TGS data is shown as well.

## Methods

### Overview of RegCloser

**Fig. 1:**
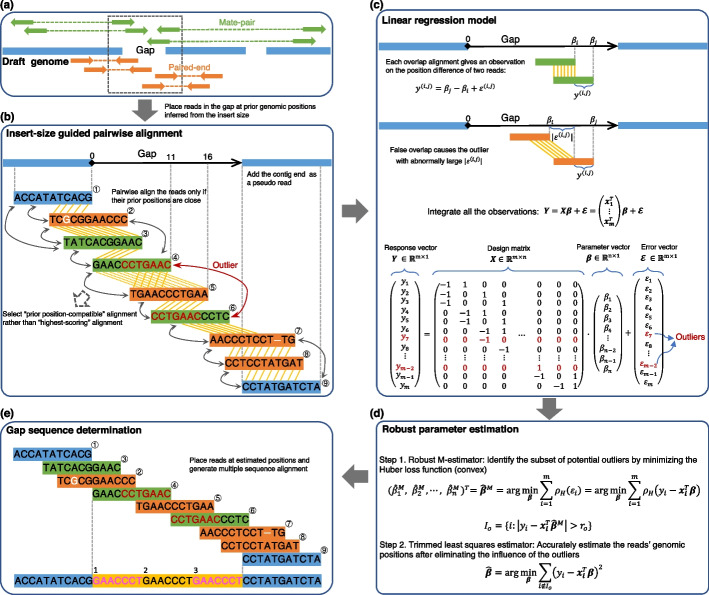
Pipeline of RegCloser. **a** Align the input paired-end or mate-pair reads onto the draft genome, and collect the reads falling in the gap regions. **b** An illustrative example of insert-size guided pairwise alignment for overlap detection in a gap containing a triple tandem repeat ‘GAACCCT’. First, on the axis corresponding to the DNA sequence in the gap, the collected reads are placed at their prior positions, which are inferred from their mate position and the insert size. The prior positions of reads ④ and ⑤ are 11 and 16. Notably, two pseudo reads, ① and ⑨, generated from the contig ends flanking the gap are added. Then, a pair of reads are aligned only when their prior positions are close, taking into account the variation of insert sizes. If an alignment is statistically significant, it is marked by yellow parallel lines between bases as well as by double-headed arrows between reads. The tandem repeat causes a false overlap between reads ④ and ⑥, as indicated by the red double-headed arrow, resulting in an outlier in the latter regression model. The repeat also leads to two different significant alignments between reads ④ and ⑤, respectively marked by the yellow solid and dotted lines. In this case, RegCloser will select the alignment more compatible with the prior positions (solid line), rather than the highest-scoring one (dotted line). **c** The linear regression model of genome assembly. The reads’ real positions on the gap axis are represented as parameters ${\beta }_{i}\ (1\le i\le n)$ to be estimated. Each detected overlap between reads $i$ and $j$ provides an observation on the difference between ${\beta }_{i}$ and ${\beta }_{j}$: ${y}^{(i,j)}={\beta }_{j}-{\beta }_{i}+{\varepsilon }^{(i,j)}$. ${\varepsilon }^{(i,j)}$ is the observational error, which is normally caused by sequencing errors on the DNA fragment between ${\beta }_{i}$ and ${\beta }_{j}$. False overlaps cause outliers, which have abnormally large $|{\varepsilon }^{\left(i,j\right)}|$. All the observations in a gap are integrated into the matrix form ${\varvec{Y}}={\varvec{X}}{\varvec{\beta}}+{\varvec{\varepsilon}}$. **d** A two-step robust regression estimation of the model parameters. ${\rho }_{H}$ is the Huber loss function. ${I}_{o}$ is the index set of observations with large residuals and identified as potential outliers. **e** Generate multiple sequence alignment of the collected reads by their estimated positions, and determine the gap sequence as the consensus between the two pseudo reads ① and ⑨. As a result, the triple tandem repeat is recovered by RegCloser

The scheme of RegCloser is illustrated in Fig. 1. RegCloser starts off by mapping input reads, SGS paired-end or mate-pair reads, onto the draft genome. Then, it collects reads from each gap region (Fig. 1a), and performs local assembly to close gaps (Fig. 1b–e). RegCloser tackles the problem of false overlaps resulted from repeats or chimeric reads by two key ideas. First, in the context of gap closing, each read from the gap region has a prior genomic position inferred from the insert size. Rather than an all-against-all pairwise alignment, RegCloser utilizes the prior positions to guide pairwise alignment (Fig. 1b) for overlap detection. In this way, false overlaps in the collecting step are substantially reduced. Second, RegCloser adopts a robust regression approach to estimate the reads’ genomic positions, which can detect the remaining false overlaps and eliminate their influences (Fig. 1c, d). Then, the reads are placed at their estimated positions in the gap, resulting in a multiple sequence alignment on which the consensus sequence in the gap is determined (Fig. 1e).

### Preprocessing

In the preprocessing stage, RegCloser first maps paired sequencing reads onto the draft genome using BWA [12]. Then, the mapped positions of the paired reads on adjacent contigs are used to estimate gap sizes. Next, RegCloser collects reads that are inferred to originate from the gap regions (Fig. 1a), and estimates their prior positions in the gap by the insert size. Specifically, let $\mu$ and $v$ be respectively the mean and standard deviation of the library insert size. For a read pair, if the left (respectively right) read is uniquely mapped within $\mu +3v$ bases from the left (respectively right) breakpoint of a gap, the mate read is inferred to come from the gap region with high confidence and is thus collected. In what follows, RegCloser assembles the collected reads from each gap in parallel.

### Overlap detection by insert-size guided pairwise alignment

We first set a coordinate axis along the gap from left to right, and the left breakpoint of the gap is defined as the origin (Fig. 1b, c). Then we represent the genomic position of read ${r}_{i}$ ($1\le i\le n$) by the coordinate of its ending base, denoted by ${\beta }_{i}$. Although the true value of ${\beta }_{i}$ is an unknown parameter, its prior genomic position, denoted by ${p}_{i}$, can be inferred from the insert size and the mapping position of the mate read on the flanking contig (Additional file 1: Fig. S1). The prior genomic positions guide the pairwise alignment to obtain more accurate and efficient overlap detection (Fig. 1b) in two aspects:

(i) RegCloser aligns two reads ${r}_{i}$ and ${r}_{j}$ only if their prior genomic positions are close to each other, i.e., $|{p}_{i}-{p}_{j}|<\Delta d$, where $\Delta d$ is a threshold depending on the standard deviation of the insert size (Additional file 1: Note S1). By choosing an appropriate $\Delta d$, we not only avoid false overlaps between reads that come from distant regions on the genome, but also reduce the time complexity of pairwise alignments from quadratic to linear (Additional file 1: Note S2). In the illustrative example shown in Fig. 1b, where reads ①–⑨ are placed along the gap axis at their prior genomic positions, we set $\Delta d=11$. That is, two reads are aligned only if their prior genomic positions are within a distance of 11 bp. Consequently, the false overlap “AACCC” between reads ② and ⑦ is avoided, and the total number of pairwise alignments is reduced to 14 from 36, which is required by the all-against-all strategy.

(ii) Two overlapping reads may have more than one significant alignment, particularly when they come from tandem repeats. RegCloser implements an extended Smith & Waterman algorithm [13] to find all the significant overlap patterns between two reads, and selects the one most compatible with the reads’ prior genomic positions. In the example shown in Fig. 1b, the two reads ④ and ⑤ actually come from a triple tandem repeat and can be aligned in two ways. RegCloser selects the correct alignment indicated by the solid line, which is closer to the relative position of ${p}_{4}$ and ${p}_{5}$, rather than the dotted one, even though it has a higher alignment score.

A real example in “Section RegCloser’s rationale of resolving the tandem repeat” will show that the above insert-size guided pairwise alignment indeed greatly reduces false overlaps.

### Representation of detected overlaps by linear regression model

In the coordinate axis along the gap, we aim to solve the true values of the reads’ genomic positions, ${\beta }_{i}$ ($1\le i\le n$), to form a layout. If a pair of reads ${r}_{i}$ and ${r}_{j}$ overlap, the alignment provides an observation on the difference of ${\beta }_{i}$ and ${\beta }_{j}$. Denote the observed genomic distance from ${r}_{i}$ to ${r}_{j}$ as ${y}^{\left(i,j\right)}$ (Fig. 1c), then

1 $$y^{{\left( {i,j} \right)}} = \beta_{j} - \beta_{i} + \varepsilon^{{\left( {i,j} \right)}} ,$$

where ${\varepsilon }^{(i,j)}$ denotes the observational error. In normal cases, $\{{\varepsilon }^{\left(i,j\right)}\}$ are the random errors caused by sequencing, and are mostly zeros for Illumina short reads (Additional file 1: Note S3). In the case of a false overlap, $|{\varepsilon }^{\left(i,j\right)}|$ will be abnormally large, and the observation will be regarded as an outlier (Fig. 1c). For example, reads ④ and ⑥ in Fig. 1e come from different repeat units, so a false overlap is detected between them, causing an outlier marked by the red double-headed arrow in Fig. 1b.

Supposing totally $m$ overlaps are detected in the gap, we integrate them into one linear regression model in the matrix form as below,

2 $${\varvec{Y}} = \user2{X\beta } + {\varvec{\varepsilon}} = \left( {\begin{array}{*{20}c} {\begin{array}{*{20}c} {{\varvec{x}}_{1}^{T} } \\ {{\varvec{x}}_{2}^{T} } \\ \end{array} } \\ {\begin{array}{*{20}c} \vdots \\ {{\varvec{x}}_{{\varvec{m}}}^{T} } \\ \end{array} } \\ \end{array} } \right){\varvec{\beta}} + {\varvec{\varepsilon}},$$

where ${\varvec{\beta}}={({\beta }_{1}, {\beta }_{2}, \cdots , {\beta }_{n})}^{T}$ denotes the vector of reads’ genomic positions; ${\varvec{X}}={({{\varvec{x}}}_{1}, {{\varvec{x}}}_{2}, \cdots , {{\varvec{x}}}_{{\varvec{m}}})}^{T}\in {\mathbb{R}}^{m\times n}$ denotes the design matrix, which is sparse as illustrated in Fig. 1c; each row of ${\varvec{X}}$ has only two non-zero elements, − 1 and 1, which are signed indicators of two overlapping reads. The corresponding term in ${\varvec{Y}}$ represents their genomic distance’s observation obtained from a detected overlap as in (1); ${\varvec{Y}}={({y}_{1}, {y}_{2}, \cdots , {y}_{m})}^{T}$ denotes the vector of all the observed genomic distances. Our aim is to estimate the reads’ genomic positions ${\varvec{\beta}}$ in the linear regression model.

Note that the design matrix ${\varvec{X}}$ in (2) is actually the oriented incidence matrix of the overlap graph, where each read corresponds to a vertex and each overlap creates a directed edge connecting two vertices. The overlap graph may have multiple weakly connected components, each of which corresponds to one contig. Accordingly, we can re-arrange the rows and columns of ${\varvec{X}}$ in (2) so that it takes a block-diagonal form as below,

3 $$\left( {\begin{array}{*{20}c} {\begin{array}{*{20}c} {\begin{array}{*{20}c} {\begin{array}{*{20}c} {{\varvec{X}}^{\left[ 1 \right]} } \\ 0 \\ \end{array} } \\ {\begin{array}{*{20}c} \vdots \\ 0 \\ \end{array} } \\ \end{array} } & {\begin{array}{*{20}c} {\begin{array}{*{20}c} 0 \\ {{\varvec{X}}^{\left[ 2 \right]} } \\ \end{array} } \\ {\begin{array}{*{20}c} \vdots \\ 0 \\ \end{array} } \\ \end{array} } \\ \end{array} } & {\begin{array}{*{20}c} {\begin{array}{*{20}c} {\begin{array}{*{20}c} \cdots \\ \cdots \\ \end{array} } \\ {\begin{array}{*{20}c} \ddots \\ \cdots \\ \end{array} } \\ \end{array} } & {\begin{array}{*{20}c} {\begin{array}{*{20}c} 0 \\ 0 \\ \end{array} } \\ {\begin{array}{*{20}c} \vdots \\ {{\varvec{X}}^{{\left[ {\varvec{s}} \right]}} } \\ \end{array} } \\ \end{array} } \\ \end{array} } \\ \end{array} } \right),$$

where all the blocks except the principal diagonal are zero matrices (Additional file 1: Note S4). Each diagonal submatrix ${{\varvec{X}}}^{[{\varvec{i}}]}\in {\mathbb{R}}^{{m}_{i}\times {n}_{i}}$ ($1\le i\le s$, $\sum_{i=1}^{s}{m}_{i}=m$, $\sum_{i=1}^{s}{n}_{i}=n$) is the oriented incidence matrix of one weakly connected component, and $\mathrm{rank}({{\varvec{X}}}^{[{\varvec{i}}]}){=n}_{i}-1$. Next, we can re-arrange the rows of ${\varvec{\beta}}$, ${\varvec{Y}}$, and ${\varvec{\varepsilon}}$ in (2) in accordance with that in ${\varvec{X}}$. Consequently, model (2) is decomposed into $s$ sub-models, i.e.,

4 $$\left\{ {\begin{array}{*{20}c} {\begin{array}{*{20}c} {{\varvec{Y}}^{\left[ 1 \right]} = {\varvec{X}}^{\left[ 1 \right]} {\varvec{\beta}}^{\left[ 1 \right]} + {\varvec{\varepsilon}}^{\left[ 1 \right]} } \\ {{\varvec{Y}}^{\left[ 2 \right]} = {\varvec{X}}^{\left[ 2 \right]} {\varvec{\beta}}^{\left[ 2 \right]} + {\varvec{\varepsilon}}^{\left[ 2 \right]} } \\ \end{array} } \\ {\begin{array}{*{20}c} \vdots \\ {{\varvec{Y}}^{{\left[ {\varvec{s}} \right]}} = {\varvec{X}}^{{\left[ {\varvec{s}} \right]}} {\varvec{\beta}}^{{\left[ {\varvec{s}} \right]}} + {\varvec{\varepsilon}}^{{\left[ {\varvec{s}} \right]}} } \\ \end{array} } \\ \end{array} } \right.,$$

where $${{\varvec{\beta}}}^{[{\varvec{i}}]}\in {\mathbb{R}}^{{n}_{i}\times 1}$$, $${{\varvec{Y}}}^{[{\varvec{i}}]}\in {\mathbb{R}}^{{m}_{i}\times 1}$$, and $${{\varvec{\varepsilon}}}^{[{\varvec{i}}]}\in {\mathbb{R}}^{{m}_{i}\times 1}$$.

Each sub-model ${{\varvec{Y}}}^{[{\varvec{i}}]}={{\varvec{X}}}^{[{\varvec{i}}]}{{\varvec{\beta}}}^{[{\varvec{i}}]}+{{\varvec{\varepsilon}}}^{[{\varvec{i}}]}$ in (4) represents one weakly connected component, i.e., one contig, and the vector ${{\varvec{\beta}}}^{[{\varvec{i}}]}$ denotes the genomic positions of the reads in this contig. Since ${{\varvec{X}}}^{[{\varvec{i}}]}$ is not full-rank in column, namely, $\mathrm{rank}({{\varvec{X}}}^{[{\varvec{i}}]}){=n}_{i}-1<{n}_{i}$, we can only estimate the parameters ${{\varvec{\beta}}}^{[{\varvec{i}}]}$ in the sub-model up to a shift. Although the relative positions of reads are sufficient to determine a layout uniquely, technically we add an initial position of one read to each sub-model to make ${{\varvec{X}}}^{[{\varvec{i}}]}$ full-rank in column so that ${{\varvec{\beta}}}^{[{\varvec{i}}]}$ is identifiable (Additional file 1: Note S4).

Estimating ${\varvec{\beta}}$ in (2) is equivalent to respectively estimating ${{\varvec{\beta}}}^{[{\varvec{i}}]}$($1\le i\le s$) in (4). Hence without loss of generality, we suppose the linear model (2) represents one contig and the design matrix ${\varvec{X}}$ is full-rank in column hereafter.

It is noted that the presentation (3) and (4) is for the sake of rigorous mathematical formulation. In practice, as shown in Fig. 1b, the two pseudo reads that are cut from the flanking sequences of a gap were incorporated into overlap detection and the linear model (2). Consequently, we only need to consider the typical scenarios shown in Additional file 1: Fig. S2. First, if the two pseudo reads are contained in one component, then the gap is filled and other floating contigs are skipped. Second, if the two pseudo reads are in two components that cannot be connected, then each flanking contig is extended by the corresponding regression component. Contigs generated from regression components other than these two major ones could be from the unfilled gap, or from elsewhere due to uncertain factors such as incorrect read mapping. In the current implementation, we leave them out for the sake of reliability.

### Estimation of the reads’ genomic positions by a two-step robust regression procedure

Although the false overlaps are significantly reduced by imposing constraint on pairwise alignment, a fraction of outliers may still exist, and even a single outlier can break down the ordinary least squares (OLS) estimate [14]. Thus, RegCloser adopts a two-step robust regression procedure (Fig. 1d) to estimate the parameters ${\varvec{\beta}}$ in (2). Note that we first relax ${\varvec{\beta}}$ from integers to be continuous values.

**Step 1:** RegCloser detects outliers using the robust M-estimate [11]. That is, minimize the sum of a loss function over all observations as below,

5 $$\hat{\user2{\beta}}^{M} = \arg \mathop {\min }\limits_{{\varvec{\beta}}} \mathop \sum \limits_{i = 1}^{m} \rho \left( {\varepsilon_{i} } \right) = \arg \mathop {\min }\limits_{{\varvec{\beta}}} \mathop \sum \limits_{i = 1}^{m} \rho \left( {y_{i} - {\varvec{x}}_{{\varvec{i}}}^{T} {\varvec{\beta}}} \right).$$

where $\rho (\bullet )$ takes the Huber loss function,

6 $$\rho_{H} \left( \varepsilon \right) = \left\{ {\begin{array}{*{20}c} {\varepsilon^{2} /2} \\ {c\left| \varepsilon \right| - c^{2} /2} \\ \end{array} } \right.{ }\begin{array}{*{20}c} {\left| \varepsilon \right| \le c} \\ {\left| \varepsilon \right| > c} \\ \end{array} .$$

**Fig. 2:**
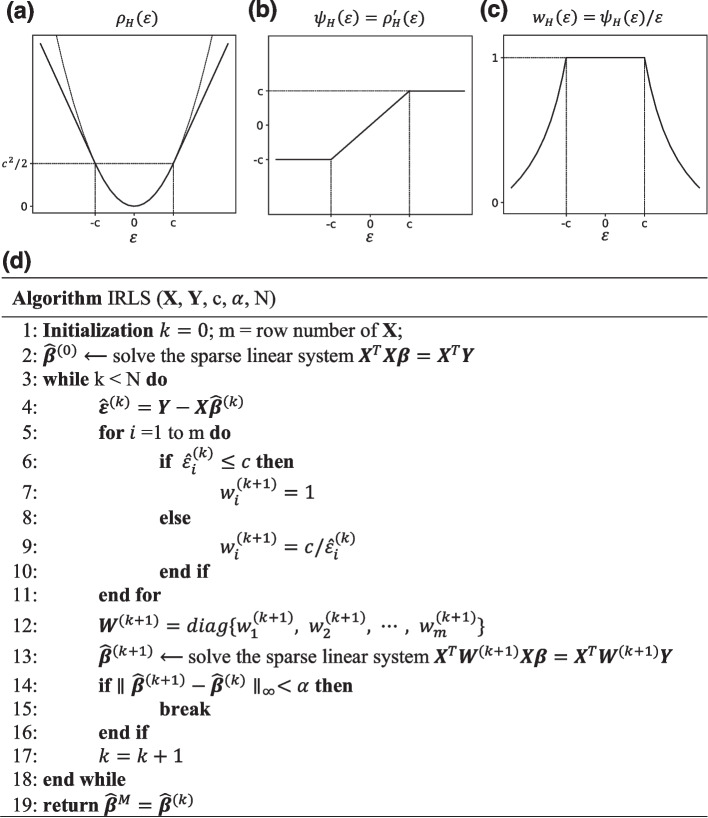
IRLS algorithm for computing the robust M-estimate. **a** The Huber loss function used in the M-estimation. Since it is convex, any local optimum is global. **b** The Huber $\psi$-function, namely, the derivative of the Huber loss function. It shows the influence of any outlier is bounded. **c** The Huber weight function. Beyond a threshold, the weight of an observation drops gradually as the error goes to large. **d** Pseudocode of the iteratively reweighted least squares (IRLS) algorithm. The inputs comprise the design matrix ${\varvec{X}}$, response vector ${\varvec{Y}}$ of the linear regression model, the tuning constant $c$, the convergence threshold $\alpha$, and the iteration limit $N$. The output is the robust M-estimate of the model parameters, which minimizes the Huber loss function

It is noted ${\rho }_{H}\left(\varepsilon \right)$ equals to the loss of squares when the observational error is small ($|\varepsilon |\le c$). But for large errors, ${\rho }_{H}\left(\varepsilon \right)$ takes absolute error loss instead (Fig. 2a) to avoid being overly influenced by the outliers. The value $c$ in (6) is a tuning constant balancing robustness and statistical efficiency. Smaller values of $c$ produce more resistance to outliers, but at the expense of larger variance of the estimate [11]. It is set to 2 by default in RegCloser.

The robustness of the M-estimate can be quantified by the influence function (IF), which measures the effect of infinitesimal perturbation of one data point on the estimate [11]. The influence function of the M-estimate (5) at an observation $({{\varvec{x}}}_{0},{y}_{0})$ is given by

7 $${\text{IF}}\left( {{\varvec{x}}_{0} ,y_{0} } \right) = \left( {\frac{{\psi_{H} \left( {y_{0} - {\varvec{x}}_{0}^{T} {\varvec{\beta}}} \right)}}{{{\text{E}}[\psi_{H}^{^{\prime}} \left( \varepsilon \right)]}}} \right)\left( {{\text{E}}\left[ {{\varvec{xx}}^{T} } \right]^{{.^{ - 1} }} {\varvec{x}}_{0} }\right),$$

where ${\psi }_{H}(\bullet )$ is the derivative of the Huber loss function (6). The first factor of (7) measures the influence from the *y*-direction, and it is bounded since ${\psi }_{H}(\bullet )$ is bounded (Fig. 2b). The second factor of (7) measures the influence from the ${\varvec{x}}$-direction, and it is also bounded since ${{\varvec{x}}}_{0}$ only consists of 0 and $\pm 1$. Putting together, the influence function (7) is bounded so that any outlier would not deviate the estimate from the true values much.

Yet we can further improve the accuracy of estimate by completely excluding the outliers. Denote the observations with large residuals by

8 $$I_{o} = \{ i:\left| {y_{i} - {\varvec{x}}_{{\varvec{i}}}^{T} \hat{\user2{\beta }}^{M} } \right| > r_{o} \} ,$$

and they are identified as potential outliers. The value ${r}_{o}$ in (8) is the residual threshold with a default value 10. A smaller value of ${r}_{o}$ gives more protection on robustness at the risk of losing some contiguity of the assembly.

**Step 2:** RegCloser solves the OLS estimate on the observations excluding the potential outliers, i.e.,

9 $$\hat{\user2{\beta }} = \arg \mathop {\min }\limits_{{\varvec{\beta}}} \mathop \sum \limits_{{i \notin I_{o} }} \left( {y_{i} - {\varvec{x}}_{{\varvec{i}}}^{T} {\varvec{\beta}}} \right)^{2} .$$

Note that one contig may be further decomposed into several contigs after excluding the potential outliers. We deal with each contig separately as described in “Section Representation of detected overlaps by linear regression model”. Finally, RegCloser obtains the estimated genomic positions of reads by rounding off ${\widehat{\beta }}_{i}$ ($1\le i\le n$) to the nearest integers. Placing the reads at their estimated positions on the coordinate axis forms a layout. In “Section RegCloser’s rationale of resolving the tandem repeat”, a real example will show the superiority of the two-step robust regression procedure over the direct OLS regression in terms of the layout quality.

### Solving the robust M-estimate by an iteratively reweighted least squares (IRLS) algorithm

In Step 1 of the two-step robust regression, an IRLS algorithm (Fig. 2d) is used to solve the robust M-estimate, i.e., the minimum of the objective function in (5). The IRLS algorithm starts with an initial estimate ${\hat{{\varvec{\beta}}}}^{(0)}$, such as the OLS estimate. Then it iteratively computes the weighted least squares (WLS) estimate as follows,

10 $${\hat{{\varvec{\beta}}}}^{(k+1)}=\mathrm{arg}\underset{{\varvec{\beta}}}{\mathrm{min}}\sum_{i=1}^{m}{w}_{i}^{(k+1)}{\left({y}_{i}-{{\varvec{x}}}_{{\varvec{i}}}^{T}{\varvec{\beta}}\right)}^{2},$$

until ${\parallel {\hat{{\varvec{\beta}}}}^{\left(k+1\right)}-{\hat{{\varvec{\beta}}}}^{(k)}\parallel }_{\infty }<\alpha$ ($\alpha =2$ by default), where ${\parallel \bullet \parallel }_{\infty }$ denotes the infinite norm. ${w}_{i}^{(k+1)}$ is the weight assigned to the $i$-th observation, and relies on the residual from the previous iteration. Specifically, ${w}_{i}^{(k+1)}={w}_{H}({\hat{\varepsilon }}_{i}^{(k)})$, where ${\hat{\varepsilon }}_{i}^{(k)}={y}_{i}-{{\varvec{x}}}_{{\varvec{i}}}^{T}{\hat{{\varvec{\beta}}}}^{(k)}$ is the residual, and ${w}_{H}(\bullet )$ is the weight function induced by the Huber loss function, i.e.,

11 $${w}_{H}\left(\varepsilon \right)=\frac{{\rho }_{H}^{\mathrm{^{\prime}}}(\varepsilon )}{\varepsilon }=\frac{{\psi }_{H}\left(\varepsilon \right)}{\varepsilon }=\left\{\begin{array}{c}1\\ c\ /\left|\varepsilon \right|\end{array}\right. \begin{array}{c}|\varepsilon |\le c\\ \left|\varepsilon \right|>c\end{array}.$$

When the residuals are small ($\le c$), the weights for the observations are 1. When the residual exceeds the threshold $c$, the weight gradually drops down (Fig. 2c). Intuitively, the IRLS algorithm assigns outliers small weights to reduce their influences on the M-estimate. Since the Huber loss function is convex and differentiable, the IRLS algorithm converges to the global minimum [15].

In each iteration of IRLS, solving the WLS estimate in (10) is equivalent to solving a system of linear equations, i.e.,

12 $${\varvec{X}}^{T} {\varvec{W}}^{\left( k \right)} \user2{X\beta } = {\varvec{X}}^{T} {\varvec{W}}^{\left( k \right)} {\varvec{Y}}.$$

The coefficient matrix ${{\varvec{X}}}^{T}{{\varvec{W}}}^{\left(k\right)}{\varvec{X}}$ is highly sparse (Additional file 1: Note S5). A series of efficient numerical algorithms have been developed for solving this kind of sparse linear systems. In RegCloser, a kind of accelerated restarted Krylov subspace method, LGMRES [16], is used to solve (12), and the matrix manipulation is implemented in the sparse format for efficiency.

When the input includes multiple libraries with different insert sizes, RegCloser has an option ‘-w’ that assigns an initial weight to each overlap observation, which corresponds to a detected overlap. The initial weight depends on the standard deviation of the library where the overlapping reads come from. The smaller standard deviation induces a larger weight.

### Gap sequence determination

For each layout of reads, a multiple sequence alignment is constructed by sequentially aligning the next read with the current alignment. A consensus is determined by selecting the nucleotide at each base site with the largest Bayesian posterior probability (Additional file 1: Note S6). The probabilities are converted into Phred scores and output to the fastq file with the nucleotide sequence. Finally, the two pseudo reads, which are cut from the flanking sequences of a gap in the overlap stage (Fig. 1b), are used to anchor the consensus into the gap (Additional file 1: Fig. S2). If the two pseudo reads are contained in the layout, the consensus sequence between them is taken to close the gap. Else if only one of the two pseudo reads is contained in the layout, RegCloser takes the consensus sequence after the left pseudo read to extend the left-flanking contig, or the consensus sequence before the right pseudo read to extend the right-flanking contig.

## Results

### Benchmarking on a GAGE dataset

We compared RegCloser with GapCloser, GapFiller, Sealer, and Phrap on the *Staphylococcus aureus* sequencing dataset from GAGE [17]. A draft assembly was generated by SOAPdenovo2 (version 2.04) [7]. A paired-end library whose insert size has an average of 415 bp and a standard deviation of 105 bp was added for closing gaps on the draft genome. The density plot of its insert size distribution is shown in Additional file 1: Fig. S3a. The read length was 101 bp and the read coverage was 84X. Quality assessment was performed through comparison with the known reference genome using QUAST (version 5.2.0) [18]. The results are shown in Additional file 1: Table S1. Sealer did not finish after running for more than 72 h. RegCloser achieved the largest contig N50 with the fewest mis-assemblies and local mis-assemblies. GapCloser achieved the largest genome fraction (98.927%) but RegCloser is close to it (98.917%). The command lines for running the five methods are provided in Additional file 1: Note S7, and their runtime and memory usage are listed in Additional file 1: Table S5.

**Fig. 3:**
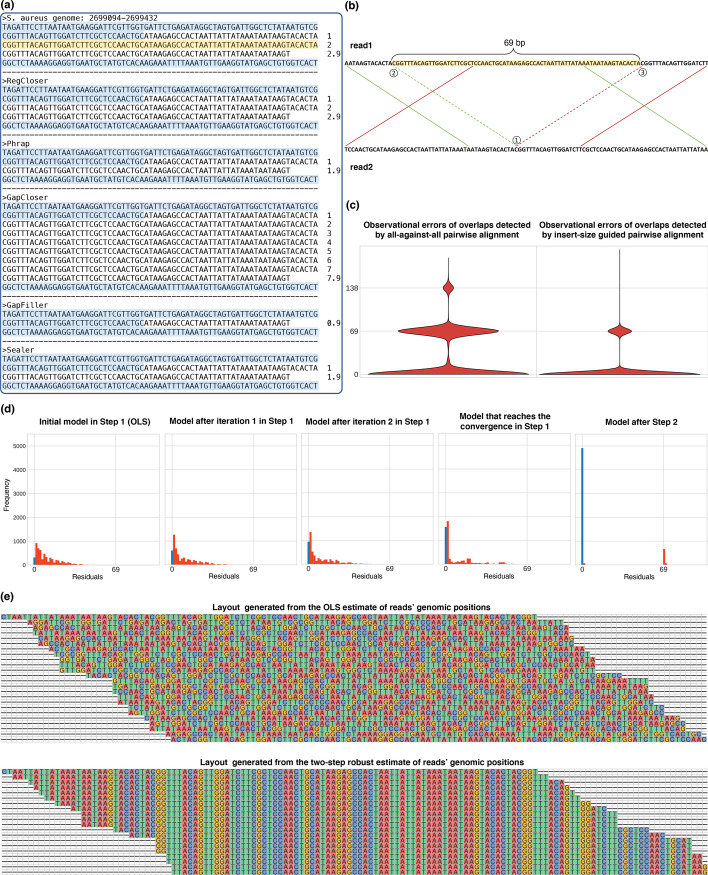
Closing a gap containing a tandem repeat. **a** Comparison of the closing results from five methods on a gap containing a triple tandem repeat. The repeat unit is shaded in yellow, and the copy number is 2.9. The gap starts at the middle of the first copy and ends at the repeat end. The sequences flanking the gap are shaded in blue. Existing tools including Phrap, GapCloser, GapFiller, and Sealer make mistakes on the copy number; only RegCloser resolves the tandem repeat correctly. **b** An illustration of the two different alignments between two reads from the tandem repeat. The position ① is mapped by the green and red alignments respectively to positions ② and ③, which differ in a shift of 69 bp, i.e., the size of the repeat unit. **c** Violin plots for the observational errors of overlaps detected by all-against-all pairwise alignment (left) versus by insert-size guided pairwise alignment (right). The observational error of an overlap between reads $i$ and $j$ refers to the difference between ${y}^{(i,j)}$ and (${\beta }_{j}-{\beta }_{i}$), where ${\beta }_{i}$ and ${\beta }_{j}$ are true read positions. The insert-size guided strategy gets rid of the large errors around 138 bp and substantially reduces the moderate errors around 69 bp, thus generating a higher-quality dataset for the regression. **d** Variation of the residual distribution along the process of the two-step robust regression. In Step 1, as the iteration increases in the IRLS algorithm for computing the robust M-estimate, most residuals converge towards 0 bp, while a fraction of residuals are still between 0 and 69 bp. In Step 2, the read coordinates are estimated by OLS on the data excluding the subset of outliers identified in Step 1. The final residuals of all data are clustered into two peaks exactly at 0 and 69 bp, which means the outliers have been separated out. **e** Screenshots of the layouts generated respectively from the OLS estimate and the two-step robust estimate of the reads’ genomic positions. The former layout is unclean and the latter layout is well aligned

Tandem repeats are ubiquitous in both prokaryote and eukaryote genomes. They are distributed in both noncoding and coding regions, and can carry important biological functions [19]. However, tandem repeats remain challenges in DNA assembly. It is particularly difficult to resolve the copy number of a tandem repeat [1]. In the evaluation of the *S. aureus* assembly, we found a gap spanned by a tandem repeat as shown in Fig. 3a. The tandem repeat was 201 bp long, with a unit size of 69 bp repeated about 2.9 times. The gap starts from the 32nd base of the first repeat unit, and ends exactly after the whole tandem repeat. All the five methods reported that they closed the gap, and the result of Sealer was obtained by running the software on the single scaffold containing the gap. However, only RegCloser resolved the copy number accurately (Fig. 3a). GapFiller, Sealer, and Phrap underestimated the copy number, while GapCloser overestimated the copy number. The result from GapFiller had 0.9 copy, those from Sealer and Phrap both had 1.9 copies, and that from GapCloser had 7.9 copies. In the next subsection, we will dissect the procedure of RegCloser and show its rationale of resolving the tandem repeat.

### RegCloser’s rationale of resolving the tandem repeat

In essence, the difficulty of solving tandem repeats is that the reads from different repeat units are hard to discriminate. False overlaps between reads from different units can lead to incorrect copy number. The analysis of the tandem repeat example shown in Fig. 3 explains how RegCloser overcomes the difficulty efficiently.

In the overlap stage, RegCloser reduced the proportion of false overlaps significantly by insert-size guided pairwise alignment. On the one hand, rather than all-against-all pairwise alignment, RegCloser detected the overlaps only between reads whose prior positions were close, and the number of pairwise alignments were reduced from 140,185 to 39,106. Consequently, the overlaps between distant repeat units were avoided from the beginning. In other words, the large observational errors (the peak of 138 bp in Fig. 3c) were eliminated from the insert-size guided overlap results (the right panel in Fig. 3c). On the other hand, when two reads from adjoining repeat units are aligned in two ways (see the example in Fig. 3b), RegCloser selected the alignment closer to the prior genomic positions. In this way, the middle observational errors (the peak of 69 bp in Fig. 3c) were further reduced substantially, with the proportion from $34.16\mathrm{\%}$ to $12.97\mathrm{\%}$. Overall, the proportion of nonzero observational errors was reduced from $48.38\mathrm{\%}$ to $14.40\mathrm{\%}$.

In the robust regression stage, RegCloser detected and eliminated the influence of the remaining false overlaps. Figure 3d shows the variation of the residual distribution along the process of the two-step robust regression. In Step 1, the IRLS algorithm started with the OLS estimate, and converged after 8 iterations. The proportion of zero residuals increased from $5.54\mathrm{\%}$ to $27.20\mathrm{\%}$, and the proportion of residuals no larger than $2$ bp increased from 21.56% to 58.73%. The overlaps with residuals larger than $10$ bp were identified as potential outliers. In Step 2, the read positions were again estimated by OLS on the data excluding the subset of potential outliers. The proportion of zero residuals further increased from $27.20\mathrm{\%}$ to 85.60 $\mathrm{\%}$, and the residuals were clustered into two peaks respectively at 0 and 69 bp (the last subplot in Fig. 3d). As illustrated in Fig. 3e, the layout resulted from the OLS estimate was full of misplacement. In contrast, the two-step robust regression in RegCloser produced a well aligned layout.

### Complete and accurate reconstruction of *E. coli* genome with simulated data

We also tested the performance of RegCloser on simulated data. Six Illumina libraries were simulated from the *Escherichia coli* reference genome (strain K-12 MG1655) using ART (version 2.5.8) [20]. The detailed information of the six libraries is listed in Additional file 1: Table S2, and the density plots of their insert size distributions are shown in Additional file 1: Fig. S3b.

A draft genome was first assembled using SOAPdenovo2 (version 2.04). The draft genome contained only one scaffold of 4,637,015 bp with 136 gaps. Five libraries with insert sizes of 300, 500, 800, 2 k, and 5 k were used to close the gaps. We ran RegCloser in an iterative way that the output genome served as the input of the next iteration. Merely after two iterations the 136 gaps were fully closed. The gap-closed genome was evaluated by QUAST (version 5.2.0). No mis-assemblies or local mis-assemblies were found, and the genome fraction was 100%. That is, a complete and accurate *E. coli* genome was reconstructed.

We compared the performance with the other four gap-closing methods. Similarly, they were run in an iterative way until no more gaps could be closed. The command lines are provided in Additional file 1: Note S7, and the runtime and memory usage are listed in Additional file 1: Table S5. The results are shown in Table 1. Only Sealer yielded one mis-assembly, while all the four methods yielded multiple local mis-assemblies. Their genome fractions were all lower than 100%. Even though GapCloser produced one contig, it contained the most local mis-assemblies. To further validate how many gaps were correctly closed, we aligned each “closed” gap sequence to the “true” gap sequence. If they were well aligned (by default, 15 bp soft-clip on both ends are allowed), the gap would be viewed as correctly closed. Table 1 demonstrates that RegCloser correctly closed all the 136 gaps, followed by GapCloser with 112/136, Phrap with 108/136, GapFiller with 103/136, and Sealer with 57/136.

**Table 1:** Comparison of the five methods on the *E. coli* simulation data

Methods	Draft	GapCloser	GapFiller	Sealer	Phrap	RegCloser
Contig length	4,531,075	4,642,829	4,631,201	4,542,679	4,641,796	4,641,652
Contig number	137	1	26	59	7	1
Contig N50	78,557	4,642,829	331,261	174,037	1,334,246	4,641,652
Genome fraction	97.532%	99.924%	99.687%	97.851%	99.892%	**100%**
# mis-assemblies	0	**0**	**0**	1	**0**	**0**
# local mis-assemblies	0	25	7	20	23	**0**
# mismatches	0	245	**43**	55	313	51
# indels	0	32	13	17	42	**12**
# closed gaps (# total gaps = 136)		**136**	111	78	130	**136**
# correctly closed gaps		112	103	57	108	**136**
# closed TRs (# total TRs = 26)		**26**	12	25	24	**26**
# correctly closed TRs		8	4	6	6	**26**
# incorrectly closed TRs/ # incorrectly closed gaps		18/24	8/8	19/21	18/22	**0/0**

The assemblies are aligned to the reference genome using QUAST 5.2.0. Genome fraction is the percentage of the reference genome covered by assembled contigs. Mis-assemblies are locations on assembled contigs where the left and right flanking sequences align over 1 kb away, or they overlap by > 1 kb, or they align on opposite strands. Local mis-assemblies are positions on contigs where the flanking sequences have a gap or overlap < 1 kbp and > 80 bp on the same strand of the reference. The best values of each quality metric are highlighted in bold. RegCloser correctly closes all the 136 gaps including the 26 tandem repeat (TR)-related gaps, and leads to a complete genome with 100% genome fraction and no mis-assemblies or local mis-assemblies. For the other four methods, the TR-related gaps account for most of the incorrectly closed gaps

We paid special attention to the correctness of the tandem repeat (TR)-related gap closing. We first used the program Tandem Repeats Finder [21] to locate tandem repeats on the reference genome, and found 26 out of the 136 gap regions intersected with certain tandem repeats. Furthermore, we found these tandem repeats accounted for most of the incorrectly closed gaps by the four methods. Specifically, 100%, $90.48\mathrm{\%}$, $81.82\mathrm{\%}$, and $75\mathrm{\%}$ of the total gaps incorrectly closed by GapFiller, Sealer, Phrap and GapCloser were TR-related respectively (Table 1).

The detailed information of the 26 TR-related gaps and their closure results are listed in Additional file 1: Table S3. RegCloser correctly closed all the 26 TR-related gaps, followed by GapCloser with 8, Phrap with 6, Sealer with 6, and GapFiller with 4. These simulation results demonstrate that RegCloser outperformed other methods, especially in resolving tandem repeats.

### Closing gaps of the plateau zokor draft genome that has been improved by long reads

To understand how the plateau zokor (*Myospalax baileyi*) adapts to the environment of high altitude and low oxygen, our collaborators from Kunming Institute of Zoology, CAS, initiated a sequencing project of plateau zokor, the complete genome size of which is about 3.17 Gbp [22]. The sequence data contain 22 Illumina libraries with insert sizes ranging from 180 bp to 10 kbp, and the total sequence coverage is 130.5 X. In addition, 10X PacBio long reads were sequenced [23]. The published draft genome was initially assembled with the Illumina short reads using SOAPdenovo2, and then was improved with the PacBio long reads using PBJelly. However, the improved genome still contained 99,434 gaps.

We run RegCloser and the other four methods to close the gaps by two rounds, and the command lines are provided in Additional file 1: Note S7. The results are shown in Table 2. In the first round, three paired-end libraries with insert sizes of 300, 500, and 800 bp were used for gap closing. In the second round, a mate-pair library with a long insert size of 3000 bp was added. The detailed information of the four libraries is provided in Additional file 1: Table S4, and the density plots of their insert size distributions are shown in Additional file 1: Fig. S3c. The runtime and memory usage of the five methods are listed in Additional file 1: Table S5.

**Table 2:** Comparison of the five methods on closing gaps of the plateau zokor draft genome

Methods	Draft	GapCloser	GapFiller	Sealer	Phrap	RegCloser
**3 paired-end libraries**						
Contig length	2,527,528,914	2,538,745,631	2,530,456,764	2,528,744,942	2,528,100,353	2,527,758,194
Contig number	113,835	97,906	96,181	102,260	71,066	69,093
Contig N50 (bp)	67,378	76,359	88,200	73,199	130,114	**143,871**
# closed gaps		15,929	17,654	11,575	42,769	**44,742**
BUSCO	95.1%	**95.2%**	95.1%	**95.2%**	**95.2%**	**95.2%**
Mapping rate	79.1%	79.4%	79.3%	79.2%	79.7%	**79.9%**
**3 paired-end + 1 mate-pair libraries**						
Contig length	2,527,528,914	2,541,442,324	2,532,550,654	2,528,949,290	2,526,621,405	2,524,372,994
Contig number	113,835	96,437	92,755	100,611	65,445	62,766
Contig N50 (bp)	67,378	77,295	93,380	74,852	146,599	**173,542**
# closed gaps		17,398	21,080	13,224	48,390	**51,069**
BUSCO	95.1%	**95.2%**	95.1%	**95.2%**	**95.2%**	**95.2%**
Mapping rate	79.1%	79.5%	79.4%	79.2%	79.7%	**80.1%**

The draft genome is initially assembled with high coverage short reads, and then improved with long reads. Two rounds of gap closing are performed. In the first round, three paired-end libraries with insert sizes of 300, 500, and 800 bp are used. In the second round, a mate-pair library with a long insert size of 3000 bp is added. Mapping rate is measured by mapping the reads of an independent library to the assembly. Contig N50 assesses the contiguity of genome assemblies. BUSCO and mapping rate assess the completeness of genome assemblies. The best values of each quality metric are highlighted in bold. After both rounds, RegCloser closes the most gaps, and achieves the highest contig N50, BUSCO, and mapping rate

After the first round, RegCloser closed 44,742 (45.0%) gaps, and the contig N50 increased from 67.4 kbp to 143.9 kbp, exceeding all the other four methods. After the second round, RegCloser closed 6,327 more gaps, and the contig N50 further increased to 173.5 kbp. We used REAPR (version 1.0.18) [24] to identify potential assembly mistakes, at which the genome would be broken. The broken contig N50 for RegCloser was 156.3 kbp, even outperforming the unbroken contig N50 for the other four methods (Table 2). It indicated RegCloser achieved the highest contiguity with high accuracy.

BUSCO (version 5.4.2) [25] was used to evaluate the completeness of assemblies with conserved single-copy orthologs in Glires. After both rounds, the BUSCO value increased from 95.1% to 95.2% for all the methods except GapFiller. Alternatively, the mapping rate of reads also measures the completeness of genome assemblies. We used the mapping tool, SEME [26] with a strict criterion, to map the reads of an independent library to the assemblies. After both rounds, RegCloser achieved the highest mapping rate (Table 2), which increased from 79.1% to 79.9% after the first round and further increased to 80.1% after the second round.

RegCloser provides an iterative scaffolding and gap closing mode like that in BAUM [27]. The mode is specified by the option “-rs” (re-scaffolding). It uses the existing scaffolding tool to re-build scaffolds before gap closing in every iteration. In this mode, contig extension and gap closing provide more information for constructing longer and more accurate scaffolds, which further benefits the gap closing in the next iteration. We successively input the 7 libraries with insert sizes of respectively 300, 500, 800, 3 k, 5 k, 8 k, and 10 k bp, and ran 7 iterations in the re-scaffolding mode. The detailed information of the 7 libraries is provided in Additional file 1: Table S4, and the density plots of their insert size distributions are shown in Additional file 1: Fig. S3c. After 7 iterations, the final contig N50 reached 204 kbp, more than 3 folds of that in the published draft. Even after broken by REAPR with both short and long insert size libraries, the contig N50 still remained 179 kbp.

### The robust regression approach to generating an optimal layout of TGS long reads

We tested the applicability of the robust regression approach to layout generation in de novo assembly of long reads using a PacBio HiFi dataset of *E. coli*. The read length ranges from 10 to 17 kbp and the sequence coverage utilized in this assembly case was about 20X. To detect overlaps, we implemented an all-against-all pairwise alignment by BLASR [28], restricting the hanging-out length to a maximum of 50 bp. Then we filtered out the suspicious chimeric reads using the information of the detected overlaps. In de novo assembly, the read orientations, namely, the strands from which the reads originate in the target genomic DNA, are unknown. Thus we first used a heuristic algorithm reported in RegScaf [29] to orientate all reads, *c.f.* Additional file 1: Note S8.

**Fig. 4:**
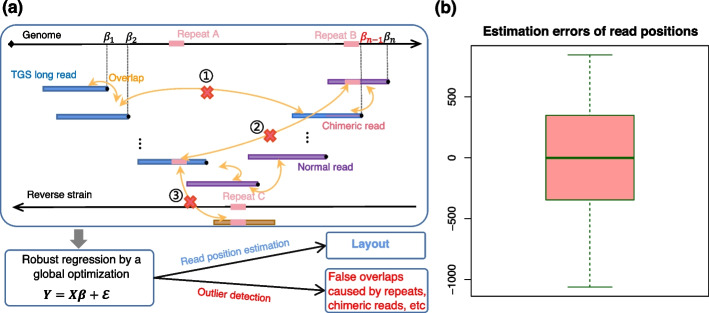
Generating an optimal layout of TGS long reads by the robust regression approach. **a** Illustration of the regression representation. In the linear axis corresponding to the real genome, the position of each TGS long read is represented by a parameter ${\beta }_{i}$ to be estimated. Each overlap, marked by the yellow double-headed arrow, provides an observation on the difference between two reads’ positions. However, chimeric reads, as well as repeats from distant regions in either the same or reverse strain of the genome, will bring in false overlaps, as those marked by red crossings, where ① indicates a false overlap caused by a chimeric read, ② indicates one caused by a repeat in the same strain, and ③ indicates one caused by a repeat in the reverse strain. All the overlap observations are integrated into the linear regression model ${\varvec{Y}}={\varvec{X}}{\varvec{\beta}}+{\varvec{\varepsilon}}$. Then the two-step robust regression procedure gives a globally optimal estimate of the read positions, which lead to a layout. Meanwhile, it detects the outliers, which correspond to the false overlaps. **b** Boxplot of the differences between the estimated and true positions of the reads in the layout that was generated by RegCloser for de novo assembly of the *E. coli* genome using a HiFi dataset

After orientating reads, we considered only one of the DNA double strands, and all reads from the other strand were transformed by reverse complement. Then these reads along with their orientation-supported alignments were input into the linear regression model described in “Section Representation of detected overlaps by linear regression model”, as depicted in Fig. 4a. The two-step robust regression procedure described in “Section Estimation of the reads’ genomic positions by a two-step robust regression procedure” was used to generate an optimal layout. The trimming step may split the resulted layout into multiple unconnected layouts. Next, we further constructed a layout-level graph in which each layout corresponds to a vertex and overlaps between layouts correspond to edges. Then the only path in the new graph led to the final layout. Its length was 4.6 M, nearly the length of the complete genome of *E. coli*.

We assessed the accuracy of the final layout by aligning all long reads to the reference genome and comparing their estimates in layouts with their real positions. The layout was shifted by a constant so that the median of errors is zero. Figure 4b is the box-plot of the errors of the estimated read positions. It shows that all reads were positioned near their true loci with a standard deviation of 451 bp.

## Discussion and conclusions

In this article, we propose a new local assembly approach, RegCloser, for closing gaps in draft genomes. On the one hand, it utilizes the insert size information in the SGS paired sequencing reads to guide the pairwise alignment, thereby reducing both the computing cost and ambiguities caused by repeats in overlap detection. On the other hand, RegCloser represents the detected overlaps by a linear regression model, which regards the false overlaps caused by repeats or sequencing errors as outliers. This representation transforms the sequence assembly into a statistically robust estimation problem.

Note that if we take the Hamiltonian graph representation in the OLC paradigm, finding an optimal layout in the overlap graph is NP-complete. Hitherto the existing OLC methods generate layouts by greedy search. Here with the linear regression representation, the global optimal layout under the Huber loss function is achievable by the IRLS algorithm, which is to iteratively solve the sparse system of linear equations. Statistically, the influence function, a robustness measure, of the Huber M-estimator is bounded in both ${\varvec{x}}$- and *y*-directions.

The aim and approach of RegCloser are different from those of the scaffolding method RegScaf we recently proposed [29], although the linear model is used in both problems. RegScaf focuses on the scaffolding problem, which is to order contigs and estimate gap sizes, while RegCloser focuses on the gap-closing problem, which is to locally assemble short reads into contigs. Technically, RegScaf adopts the least trimmed squares (LTS) estimator. Although no efficient algorithm for computing the exact LTS solution is available in the multiple regression, an approximation is sufficiently good for the accuracy required by scaffolding. In comparison, the layout generation in the assembly problem needs more accurate estimation of read positions. Therefore, RegCloser adopts the more efficient robust estimator, the Huber M-estimator, whose exact solution can be computed. RegCloser can be used to improve genome assemblies in combination with any scaffolding methods.

When the repeat unit size is beyond the length of sequencing reads, the repeat structure can hardly be resolved by SGS data alone. However, we demonstrated that making full use of the library insert size information combined with robust regression can resolve some tandem repeats successfully. The insert-size guided pairwise alignment helps distinguish repeats from different units, and the two-step robust regression lays out reads at the right places. Compared with existing gap-closing methods, RegCloser closes more gaps and achieves higher contiguity and completeness, especially outperforms at gaps containing tandem repeats. The simulation results and the quality assessment by REAPR indicate that the gaps closed by RegCloser are of high quality.

The results on the plateau zokor draft genome suggest that RegCloser is scalable to large genomes. The zokor draft genome was initially assembled from SGS data, then long reads were applied to improve its contig N50 to 67 kbp. RegCloser further increased the contig N50 to 204 kbp based on the iterative scaffolding and gap closing strategy, which indicates that RegCloser can still improve on the basis of the results from long reads.

Conceptually, the robust regression approach is a general assembly framework that is applicable to de novo assembly of both SGS and TGS data. We tested it on the layout generation for TGS long reads. Despite the long read length of TGS technology, issues like chimeric reads and indistinguishable repeats of a longer unit size still exist. Our results suggest that these issues can be partly solved by robust regression. Therefore, the proposed robust regression approach has a prospect to be incorporated into the layout module of long read assemblers.

## Supplementary Information

**Additional file 1. **Supplementary Figs. S1-S3, Notes S1-S8, and Tables S1-S5. 

## Data Availability

The *S. aureus* GAGE dataset is available at http://gage.cbcb.umd.edu/data. The *S. aureus* paired-end sequence data are available in the Sequence Read Archive (SRA) in National Center for Biotechnology Information (NCBI, https://www.ncbi.nlm.nih.gov) under the accession number SRR5244695. The *S. aureus* reference genome is available in the Assembly database in NCBI under the accession number GCF_000017085.1. The *E. coli* simulated sequence data can be downloaded from our github site at https://github.com/csh3/RegCloser. The *E. coli* reference genome is available in the Assembly database in NCBI under the accession number GCF_000005845.2. The plateau zokor draft genome was retrieved from the Genome Warehouse (GWH) in National Genomics Data Center (NGDC, https://ngdc.cncb.ac.cn) under the accession number GWHABJZ00000000. The raw Illumina sequence data of plateau zokor are accessible in the Genome Sequence Archive (GSA) in NGDC under accession numbers ranging from CRX003877 to CRX003883, and from CRX004479 to CRX004484. The plateau zokor genome improved by RegCloser has been deposited in the GWH in NGDC under the accession number GWHBOZX00000000. The PacBio HiFi dataset of *E. coli* is available in the SRA in NCBI under the accession number SRR10971019. The software details are listed below Project name: RegCloser Project home page: https://github.com/csh3/RegCloser Operating system: Linux Programming language: Python Other requirements: BWA, Python3 with modules os, sys, re, argparse, biopython, numpy, math, networkx, scipy, collections, datetime, multiprocessing License: GNU General Public License v3.0 only Any restrictions to use by non-academics: licence needed

## References

[1] Tørresen OK, Star B, Mier P, Andrade-Navarro MA, Bateman A, Jarnot P, Gruca A, Grynberg M, Kajava AV, Promponas VJ (2019). Tandem repeats lead to sequence assembly errors and impose multi-level challenges for genome and protein databases. Nucleic Acids Res.

[2] Bolivar-Torres HH, Marín-Paredes R, Ramos-Madrigal C, Servín-Garcidueñas LE (2022). Metagenome-assembled genome of acidibrevibacterium fodinaquatile FLA01 from fumarole sediments from the Los Azufres Geothermal Field. Microbiol Resource Announc.

[3] Tahir J, Crowhurst R, Deroles S, Hilario E, Schaffer R, Le Lievre L, Brendolise C, Chagne D, Gardiner SE, Knaebel M. First chromosome-scale assembly and deep floral-bud transcriptome of a male kiwifruit. Front Genet. 2022;961. 10.3389/fgene.2022.852161 PMC9149279 35651931

[4] Hammond SA, Warren RL, Vandervalk BP, Kucuk E, Khan H, Gibb EA, Pandoh P, Kirk H, Zhao Y, Jones M (2017). The North American bullfrog draft genome provides insight into hormonal regulation of long noncoding RNA. Nat Commun.

[5] Gold DA, Katsuki T, Li Y, Yan X, Regulski M, Ibberson D, Holstein T, Steele RE, Jacobs DK, Greenspan RJ (2019). The genome of the jellyfish Aurelia and the evolution of animal complexity. Nat Ecol Evolut.

[6] Paulino D, Warren RL, Vandervalk BP, Raymond A, Jackman SD, Birol I (2015). Sealer: a scalable gap-closing application for finishing draft genomes. BMC Bioinform.

[7] Luo R, Liu B, Xie Y, Li Z, Huang W, Yuan J, He G, Chen Y, Pan Q, Liu Y (2012). SOAPdenovo2: an empirically improved memory-efficient short-read de novo assembler. Gigascience.

[8] Boetzer M, Pirovano W (2012). Toward almost closed genomes with GapFiller. Genome Biol.

[9] Green P: PHRAP documentation. http://www.phrap.org (13 September 2022, date last accessed). 1994.

[10] Skiena SS (2008). The algorithm design manual.

[11] Maronna RA, Martin RD, Yohai VJ (2006). Robust statistics: theory and methods.

[12] Li H, Durbin R (2009). Fast and accurate short read alignment with Burrows-Wheeler transform. Bioinformatics.

[13] Waterman MS, Eggert M (1987). A new algorithm for best subsequence alignments with application to tRNA-rRNA comparisons. J Mol Biol.

[14] Yohai VJ: High breakdown-point and high efficiency robust estimates for regression. Ann Stat 1987;15(2):642–656, 615.

[15] Aftab K, Hartley R. Convergence of iteratively re-weighted least squares to robust M-estimators. In: Proceedings of the 2015 IEEE winter conference on applications of computer vision. IEEE Computer Society 2015: 480–487.

[16] Baker AH, Jessup ER, Manteuffel TA (2005). A technique for accelerating the convergence of restarted GMRES. SIAM J Matrix Anal Appl.

[17] Salzberg SL, Phillippy AM, Zimin A, Puiu D, Magoc T, Koren S, Treangen TJ, Schatz MC, Delcher AL, Roberts M (2012). GAGE: a critical evaluation of genome assemblies and assembly algorithms. Genome Res.

[18] Gurevich A, Saveliev V, Vyahhi N, Tesler G (2013). QUAST: quality assessment tool for genome assemblies. Bioinformatics.

[19] Usdin K (2008). The biological effects of simple tandem repeats: lessons from the repeat expansion diseases. Genome Res.

[20] Huang W, Li L, Myers JR, Marth GT (2012). ART: a next-generation sequencing read simulator. Bioinformatics.

[21] Benson G (1999). Tandem repeats finder: a program to analyze DNA sequences. Nucleic Acids Res.

[22] Xu D, Yang C, Shen Q, Pan S, Liu Z, Zhang T, Zhou X, Lei M, Chen P, Yang H (2021). A single mutation underlying phenotypic convergence for hypoxia adaptation on the Qinghai-Tibetan Plateau. Cell Res.

[23] Zhang T, Chen J, Zhang J, Guo YT, Zhou X, Li MW, Zheng ZZ, Zhang TZ, Murphy RW, Nevo E (2021). Phenotypic and genomic adaptations to the extremely high elevation in plateau zokor (Myospalax baileyi). Mol Ecol.

[24] Hunt M, Kikuchi T, Sanders M, Newbold C, Berriman M, Otto TD (2013). REAPR: a universal tool for genome assembly evaluation. Genome Biol.

[25] Simão FA, Waterhouse RM, Ioannidis P, Kriventseva EV, Zdobnov EM (2015). BUSCO: assessing genome assembly and annotation completeness with single-copy orthologs. Bioinformatics.

[26] Chen S, Wang A, Li LM (2013). SEME: a fast mapper of Illumina sequencing reads with statistical evaluation. J Comput Biol.

[27] Wang A, Wang Z, Li Z, Li LM (2018). BAUM: improving genome assembly by adaptive unique mapping and local overlap-layout-consensus approach. Bioinformatics.

[28] Chaisson MJ, Tesler G (2012). Mapping single molecule sequencing reads using basic local alignment with successive refinement (BLASR): application and theory. BMC Bioinformatics.

[29] Li M, Li LM (2022). RegScaf: a regression approach to scaffolding. Bioinformatics.

