# Supplementary material for: RegCloser: a robust regression approach to closing genome gaps
Source: BMC Bioinformatics. 2023 Jun 13;24:249. doi: 10.1186/s12859-023-05367-0 (PMC10265787; doi:10.1186/s12859-023-05367-0)
Supplement: Supplementary file 1 — Additional file 1. Supplementary Figs. S1-S3, Notes S1-S8, and Tables S1-S5. [file 12859_2023_5367_MOESM1_ESM.docx]

Supplementary Materials for

**RegCloser: a robust regression approach to closing genome gaps**

Shenghao Cao, Mengtian Li and Lei M. Li^*^

*Correspondence: [lilei@amss.ac.cn](mailto:lilei@amss.ac.cn)

**Contents**

[Supplementary Figures 2](#_Toc134520811)

[Figure S1. Illustration of calculating the reads’ prior genomic positions in the gap. 2](#_Toc134520812)

[Figure S2. Illustration of anchoring the gap sequence by pseudo reads. 3](#_Toc134520813)

[Figure S3. Density plots of insert size distributions of the sequencing libraries used for gap closing of the three draft genomes. 4](#_Toc134520814)

[Supplementary Notes 5](#_Toc134520815)

[Note S1. The maximum admissible distance between prior positions of two reads that are pairwise aligned 5](#_Toc134520816)

[Note S2. Linear time complexity of the insert-size guided pairwise alignment 6](#_Toc134520817)

[Note S3. Probability property of the observational error $\varepsilon^{(i,j)}$ 7](#_Toc134520818)

[Note S4. Contig decomposition of the linear regression model $\boldsymbol{Y}=\boldsymbol{X\beta}+\boldsymbol{\varepsilon}$ 8](#_Toc134520819)

[Note S5. Sparsity of the coefficient matrix $\boldsymbol{X}^{\boldsymbol{T}}\boldsymbol{W}^{\boldsymbol{(k)}}\boldsymbol{X}$ 10](#_Toc134520820)

[Note S6. Bayesian posterior probability per nucleotide type at each base site 11](#_Toc134520821)

[Note S7. Command lines used in the Results section 12](#_Toc134520822)

[Note S8. The orientating algorithm used in the layout generation of TGS long reads 13](#_Toc134520823)

[Supplementary Tables 14](#_Toc134520824)

[Table S1. Comparison of the five methods on the *S. aureus* sequencing dataset 14](#_Toc134520825)

[Table S2. Detailed information of the 6 simulation libraries of *E. coli* 15](#_Toc134520826)

[Table S3. Detailed information of the 26 TR-related gaps on the *E. coli* draft genome and their closure results from the five methods 16](#_Toc134520827)

[Table S4. Detailed information of the 7 sequencing libraries of the plateau zokor genome 17](#_Toc134520828)

[Table S5. Runtime and memory usage of the five gap-closing tools on the three data sets 18](#_Toc134520829)

# Supplementary Figures

Figure S1. Illustration of calculating the reads’ prior genomic positions in the gap. Read $r_{i}$ is collected from the gap region, and read $r_{i}^{*}$ is its mate read. A coordinate axis is set along the gap from left to right, and the left breakpoint of the gap is defined as the origin. The prior genomic position of read $r_{i}$ in the gap, denoted by $p_{i}$, is defined as the inferred coordinate of its ending base in the axis. **(a)** If $r_{i}^{*}$ aligns to the left-flanking contig of the gap, $p_{i}=\mu_{i}-a_{i}$, where $\mu_{i}$ denotes the insert size between $r_{i}$ and $r_{i}^{*}$, and $a_{i}$ denotes the mapping position of $r_{i}^{*}$ on the flanking contig. **(b)** If $r_{i}^{*}$ aligns to the right-flanking contig of the gap, $p_{i}=g-\left( \mu_{i}-a_{i} \right)+l_{i}$, where $g$ denotes the gap size and $l_{i}$ denotes the read length of $r_{i}$.

Figure S2. Illustration of anchoring the gap sequence by pseudo reads. In each gap, the reads may be assembled into more than one contig. The layout of each contig is circled by yellow. The two pseudo reads (blue ones), which are cut from the flanking sequences of the gap, are used to anchor the consensus sequence into the gap. **(a)** If the two pseudo reads are contained in one contig, the consensus sequence between them is taken to close the gap. **(b)** If the two pseudo reads are in two separate contigs, the consensus sequence after the left pseudo read is taken to extend the left-flanking contig, and the consensus sequence before the right pseudo read is taken to extend the right-flanking contig.


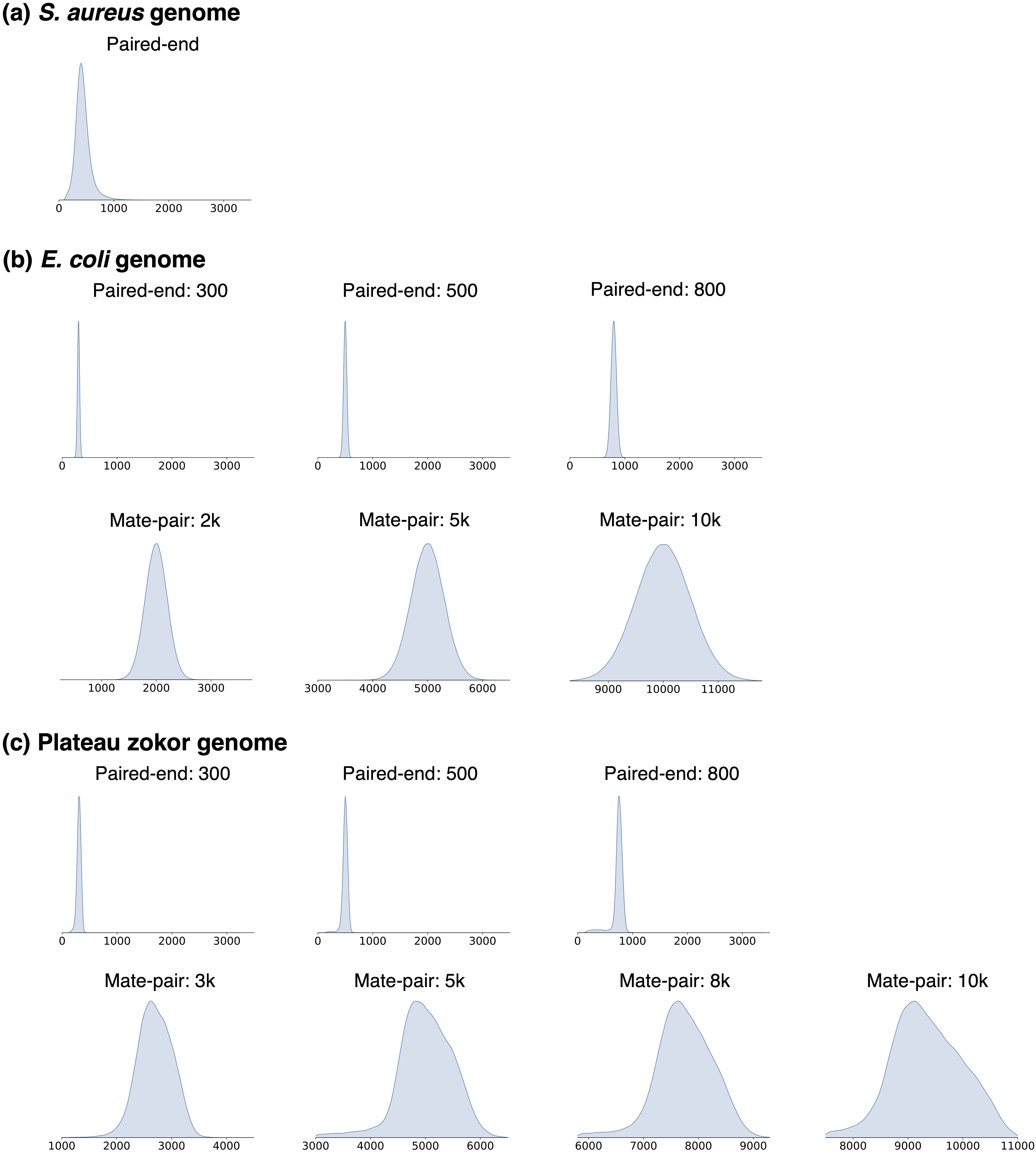


Figure S3. Density plots of insert size distributions of the sequencing libraries used for gap closing of the three draft genomes. The horizontal axis of each plot represents the insert size (base pair). The scales in the horizontal axes are the same across the plots.

# Supplementary Notes

## Note S1. The maximum admissible distance between prior positions of two reads that are pairwise aligned

For any two reads $r_{i}$ and $r_{j}$ collected in the gap, they have prior genomic positions $p_{i}$ and $p_{j}$ inferred from the insert size. Suppose the library insert size obeys the normal distribution with the standard deviation for $r_{i}$ being $\sigma_{i}$, for $r_{j}$ being $\sigma_{j}$. Denote the real genomic positions of $r_{i}$ and $r_{j}$ as $P_{i}$ and $P_{j}$, then $p_{i} \sim N\left( P_{i},\sigma_{i}^{2} \right)$ and $p_{j} \sim N\left( P_{j},\sigma_{j}^{2} \right)$. Since any two reads are independent, we have $p_{i}-p_{j} \sim N\left( P_{i}-P_{j}, \sigma^{2} \right)$, where $\sigma=\sqrt{\sigma_{i}^{2}+\sigma_{j}^{2}}$. Suppose a successful alignment should have an overlap length of at least *k* bp, i.e., $\left| P_{i}-P_{j} \right|\leq l-k$, where *l* denotes the read length.

**Proposition:** For any two overlapping reads, $\Pr\left( |p_{i}-p_{j}|\leq l-k+\sigma z_{1-\frac{\alpha}{2}} \right)\geq1-\alpha$, where $z_{1-\frac{\alpha}{2}}$ is the $1-\frac{\sigma}{2}$ quantile of the standard normal distribution.

**Proof:** Since $p_{i}-p_{j} \sim N\left( P_{i}-P_{j}, \sigma^{2} \right)$, $\Pr\left( p_{i}-p_{j}\in\left[ P_{i}-P_{j}-\sigma z_{1-\frac{\alpha}{2}}, P_{i}-P_{j}+\sigma z_{1-\frac{\alpha}{2}} \right] \right)=1-\alpha$. For any two overlapping reads, we have ${-(l-k)\leq P}_{i}-P_{j}\leq l-k$, so that $\left[ P_{i}-P_{j}-\sigma z_{1-\frac{\alpha}{2}}, P_{i}-P_{j}+\sigma z_{1-\frac{\alpha}{2}} \right]\subseteq\left[ -\left( l-k \right)-\sigma z_{1-\frac{\alpha}{2}}, l-k+\sigma z_{1-\frac{\alpha}{2}} \right]$. Therefore,

$$1-\alpha=\Pr\left( p_{i}-p_{j}\in\left[ P_{i}-P_{j}-\sigma z_{1-\frac{\alpha}{2}}, P_{i}-P_{j}+\sigma z_{1-\frac{\alpha}{2}} \right] \right)$$

$$\leq\Pr\left( p_{i}-p_{j}\in\left[ -\left( l-k \right)-\sigma z_{1-\frac{\alpha}{2}}, l-k+\sigma z_{1-\frac{\alpha}{2}} \right] \right)$$

$=\Pr\left( |p_{i}-p_{j}|\leq l-k+\sigma z_{1-\frac{\alpha}{2}} \right)$.

**Remark:** We pairwise align two reads only if $\left| p_{i}-p_{j} \right|\leq\Delta d$, where the threshold $\Delta d$ is set as $l-k+\sigma z_{1-\frac{\alpha}{2}}$ $. z_{1-\frac{\alpha}{2}}$ is the tuning parameter specified by the option ‘-S’ in the software, and its default value is 0.3, corresponding to $\alpha\approx0.76$. The above proposition means that the probability of any two overlapping reads being aligned is greater than $1-\alpha$. Supposing the read coverage is $c$, then for any one read, the probability of at least one read overlapping with it being aligned is approximately greater than $1-\alpha^{c}$. In the default setting, $1-\alpha^{c}\approx1-{0.76}^{c}$, which is greater than $0.995$ when $c\geq20.$ In a typical case of $l=100$, $k=20$, and $\sigma=50$, we have the default value of $\Delta d=100-20+50*0.3=95$.

## Note S2. Linear time complexity of the insert-size guided pairwise alignment

**Proposition:** The number of pairwise alignments guided by the insert size is linear with the read number $n$, i.e., $T(n)=\frac{\Delta d\cdot c}{l}n$, where $c$ denotes the coverage of collected reads in the gap region; $l$ denotes the read length; $\Delta d$ denotes the threshold for guiding the pairwise alignment. Two reads are aligned only if the difference of their prior genomic positions is less than $\Delta d$.

**Proof:** Suppose the collected reads are uniformly distributed in the gap region. Each read $r_{i}$ only aligns with reads falling in the interval $[p_{i}-\Delta d, p_{i}+\Delta d]$, where $p_{i}$ is the prior genomic position of $r_{i}$. Then, the number of reads aligned with $r_{i}$ is $\frac{2\Delta d\cdot c}{l}$. Therefore, the total number of pairwise alignments is $\frac{1}{2}\frac{2\Delta d\cdot c}{l}n=\frac{\Delta d\cdot c}{l}n$.

**Remark:** Let $g$ denote the gap size. Then, the read number is $n=\frac{g\cdot c}{l}$, which is proportional to the gap size $g$ when the read coverage $c$ is a constant. In this case, the number of pairwise alignments guided by the insert size, $T\left( n \right)=\frac{\Delta d\cdot c}{l}n$, is linear with the read number $n$, while the number of all-against-all pairwise alignments are $\frac{n^{2}}{2}$, quadratic with $n$. Thus, the time complexity of pairwise alignments is reduced from quadratic one to linear, and a larger gap size results in a greater reduction.

## Note S3. Probability property of the observational error $\boldsymbol{\varepsilon}^{\mathbf{(}\boldsymbol{i}\boldsymbol{,}\boldsymbol{j}\mathbf{)}}$

For each pair of overlapping reads, $\varepsilon^{(i,j)}$ equals to the number of inserted bases minus the number of deleted bases on the DNA fragment between $\beta_{i}$ and $\beta_{j}$. Thus, if no insertion or deletion (indel) errors in the sequencing reads, $\varepsilon^{(i,j)}$ would be equal to 0. Supposing the indel error rate of each base site is $r$ and the sequencing read length is $l$, then we have

$\Pr\left( \varepsilon^{(i,j)}=0 \right)\geq\left( 1-r \right)^{l}$.

Since the indel error rates of Illumina short reads are generally very low, the probability of $\varepsilon^{(i,j)}=0$ is close to 1. That is, $\varepsilon^{(i,j)}$ is mostly zero.

For HiSeq reads, it is reported that the indel error rate $r$ is normally less than 0.00003 and the read length $l$ is up to 125 bp [[1](#_ENREF_1)], i.e., $r<0.00003$ and $l\leq125$. Therefore, we have

$\Pr\left( \varepsilon^{(i,j)}=0 \right)> \left( 1-0.00003 \right)^{125}>0.996$.

For MiSeq reads, it is reported that the indel error rate $r$ is normally less than 0.00005 and the read length $l$ is up to 300 bp [[2](#_ENREF_2)], i.e., $r<0.00005$ and $l\leq300$. Therefore, we have

$\Pr\left( \varepsilon^{(i,j)}=0 \right)> \left( 1-0.00005 \right)^{300}>0.985$.

## Note S4. Contig decomposition of the linear regression model $\boldsymbol{Y}\mathbf{=}\boldsymbol{X\beta}\mathbf{+}\boldsymbol{\varepsilon}$

The detected overlaps across reads are represented by the linear regression model (2) in the main text, i.e.,

$$\boldsymbol{Y}=\boldsymbol{X\beta}+\boldsymbol{\varepsilon}=\binom{\begin{matrix} \boldsymbol{x}_{\boldsymbol{1}}^{T} \\ \boldsymbol{x}_{\boldsymbol{2}}^{T} \end{matrix}}{\begin{matrix} \vdots\\ \boldsymbol{x}_{\boldsymbol{m}}^{T} \end{matrix}}\boldsymbol{\beta}+\boldsymbol{\varepsilon}.$$

The design matrix $\boldsymbol{X}$ is the oriented incidence matrix of the overlap graph, where each read corresponds to a vertex and each overlap creates a directed edge connecting two vertices. A directed graph $G$ is called weakly connected if replacing all of its directed edges with undirected edges produces a connected undirected graph. If $G$ is not weakly connected, let $W$ be a maximal subset of vertices such that for every pair of vertices $u, v\in W$, $u$ and $v$ are connected by a path in the underlying undirected graph of $G$. Then the directed subgraph induced by $W$ is called a weakly connected component of $G$ [[3](#_ENREF_3)]. Supposing the overlap graph is decomposed into $s$ weakly connected components, then each component corresponds to one contig.

For the reads in the i-th component, we denote by $\boldsymbol{\beta}^{[\boldsymbol{i}]}={(\beta_{j_{1}^{(i)}}, \beta_{j_{2}^{(i)}}, \cdots, \beta_{j_{n_{i}}^{(i)}})}^{T}$ the reads’ corresponding parameters in $\boldsymbol{\beta}$, and by $\boldsymbol{Y}^{[\boldsymbol{i}]}={(y_{k_{1}^{(i)}}, y_{k_{2}^{(i)}}, \cdots, y_{k_{m_{i}}^{(i)}})}^{T}$ the corresponding response values in $\boldsymbol{Y}$ observed from the overlaps. Then, the overlaps of the reads in the i-th component can be represented by the sub-model,

$$\boldsymbol{Y}^{\left[ \boldsymbol{i} \right]}=\boldsymbol{X}^{\left[ \boldsymbol{i} \right]}\boldsymbol{\beta}^{\left[ \boldsymbol{i} \right]}+\boldsymbol{\varepsilon}^{\left[ \boldsymbol{i} \right]},$$

where $\boldsymbol{\varepsilon}^{[\boldsymbol{i}]}={(\varepsilon_{k_{1}^{(i)}}, \varepsilon_{k_{2}^{(i)}}, \cdots, \varepsilon_{k_{m_{i}}^{(i)}})}^{T},$ $\boldsymbol{X}^{\left[ \boldsymbol{i} \right]}\in\mathbb{R}^{m_{i}\times n_{i}}$ is the submatrix of $\boldsymbol{X}\in\mathbb{R}^{m\times n}$ as below,

$$\boldsymbol{X}^{\left[ \boldsymbol{i} \right]}=\left( \begin{matrix} \begin{matrix} \begin{matrix} \begin{matrix} X_{k_{1}^{(i)},j_{1}^{(i)}} \\ X_{k_{2}^{(i)},j_{1}^{(i)}} \end{matrix} \\ \begin{matrix} \vdots\\ X_{k_{m_{i}}^{(i)},j_{1}^{(i)}} \end{matrix} \end{matrix} & \begin{matrix} \begin{matrix} X_{k_{1}^{(i)},j_{2}^{(i)}} \\ X_{k_{2}^{(i)},j_{2}^{(i)}} \end{matrix} \\ \begin{matrix} \vdots\\ X_{k_{m_{i}}^{(i)},j_{2}^{(i)}} \end{matrix} \end{matrix} \end{matrix} & \begin{matrix} \cdots& \begin{matrix} \begin{matrix} X_{k_{1}^{(i)},j_{n_{i}}^{(i)}} \\ X_{k_{2}^{(i)},j_{n_{i}}^{(i)}} \end{matrix} \\ \begin{matrix} \vdots\\ X_{k_{m_{i}}^{(i)},j_{n_{i}}^{(i)}} \end{matrix} \end{matrix} \end{matrix} \end{matrix} \right),$$

and each row of $\boldsymbol{X}^{\left[ \boldsymbol{i} \right]}$ has only two non-zero elements -1 and 1 as in $\boldsymbol{X}$. $\boldsymbol{X}^{\left[ \boldsymbol{i} \right]}$ is the oriented incidence matrix of the i-th component.

We alter the order of the columns of $\boldsymbol{X}$ from $(1,2,\cdots,n)$ to $(j_{1}^{\left( 1 \right)},\cdots,j_{n_{1}}^{(1)},\cdots,j_{1}^{\left( s \right)},\cdots,j_{n_{s}}^{(s)})$, and the order of the rows of $\boldsymbol{X}$ from $(1,2,\cdots,m)$ to $(k_{1}^{\left( 1 \right)},\cdots,k_{m_{1}}^{(1)},\cdots,k_{1}^{\left( s \right)},\cdots,k_{m_{s}}^{(s)})$. Then, $\boldsymbol{X}$ is transformed into a partitioned matrix in the block-diagonal form as (3) in the main text, i.e.,

$\left( \begin{matrix} \begin{matrix} \begin{matrix} \begin{matrix} \boldsymbol{X}^{[\boldsymbol{1}]} \\ \boldsymbol{0} \end{matrix} \\ \begin{matrix} \vdots\\ \boldsymbol{0} \end{matrix} \end{matrix} & \begin{matrix} \begin{matrix} \boldsymbol{0} \\ \boldsymbol{X}^{[\boldsymbol{2}]} \end{matrix} \\ \begin{matrix} \vdots\\ \boldsymbol{0} \end{matrix} \end{matrix} \end{matrix} & \begin{matrix} \begin{matrix} \begin{matrix} \cdots\\ \cdots\end{matrix} \\ \begin{matrix} \ddots\\ \cdots\end{matrix} \end{matrix} & \begin{matrix} \begin{matrix} \boldsymbol{0} \\ \boldsymbol{0} \end{matrix} \\ \begin{matrix} \vdots\\ \boldsymbol{X}^{[\boldsymbol{s}]} \end{matrix} \end{matrix} \end{matrix} \end{matrix} \right)$.

We accordingly alter the order of the rows of $\boldsymbol{Y}$ and $\boldsymbol{\varepsilon}$ from $(1,2,\cdots,m)$ to $(k_{1}^{\left( 1 \right)},\cdots,k_{m_{1}}^{(1)},\cdots,k_{1}^{\left( s \right)},\cdots,k_{m_{s}}^{(s)})$, and that of $\boldsymbol{\beta}$ from $(1,2,\cdots,n)$ to $(j_{1}^{\left( 1 \right)},\cdots,j_{n_{1}}^{(1)},\cdots,j_{1}^{\left( s \right)},\cdots,j_{n_{s}}^{(s)})$. Then, the linear regression model $\boldsymbol{Y}=\boldsymbol{X\beta}+\boldsymbol{\varepsilon}$ can be re-written as below,

$$\binom{\begin{matrix} \boldsymbol{Y}^{[\boldsymbol{1}]} \\ \boldsymbol{Y}^{[\boldsymbol{2}]} \end{matrix}}{\begin{matrix} \vdots\\ \boldsymbol{Y}^{[\boldsymbol{s}]} \end{matrix}}=\left( \begin{matrix} \begin{matrix} \begin{matrix} \begin{matrix} \boldsymbol{X}^{[\boldsymbol{1}]} \\ \boldsymbol{0} \end{matrix} \\ \begin{matrix} \vdots\\ \boldsymbol{0} \end{matrix} \end{matrix} & \begin{matrix} \begin{matrix} \boldsymbol{0} \\ \boldsymbol{X}^{[\boldsymbol{2}]} \end{matrix} \\ \begin{matrix} \vdots\\ \boldsymbol{0} \end{matrix} \end{matrix} \end{matrix} & \begin{matrix} \begin{matrix} \begin{matrix} \cdots\\ \cdots\end{matrix} \\ \begin{matrix} \ddots\\ \cdots\end{matrix} \end{matrix} & \begin{matrix} \begin{matrix} \boldsymbol{0} \\ \boldsymbol{0} \end{matrix} \\ \begin{matrix} \vdots\\ \boldsymbol{X}^{[\boldsymbol{s}]} \end{matrix} \end{matrix} \end{matrix} \end{matrix} \right)\cdot\binom{\begin{matrix} \boldsymbol{\beta}^{\left[ \boldsymbol{1} \right]} \\ \boldsymbol{\beta}^{\left[ \boldsymbol{2} \right]} \end{matrix}}{\begin{matrix} \vdots\\ \boldsymbol{\beta}^{\left[ \boldsymbol{s} \right]} \end{matrix}}+\binom{\begin{matrix} \boldsymbol{\varepsilon}^{\left[ \boldsymbol{1} \right]} \\ \boldsymbol{\varepsilon}^{\left[ \boldsymbol{2} \right]} \end{matrix}}{\begin{matrix} \vdots\\ \boldsymbol{\varepsilon}^{\left[ \boldsymbol{s} \right]} \end{matrix}},$$

so that the model is decomposed into $s$ sub-models as (4) in the main text, and each sub-model corresponds to a weakly connected component, i.e., a contig.

**Proposition:** The solutions to the linear equations system $\boldsymbol{X}^{\left[ \boldsymbol{i} \right]}\boldsymbol{b}=\boldsymbol{0}$ are $\boldsymbol{b}={(\Delta,\Delta,\cdots,\Delta)}^{T}=\Delta\cdot\boldsymbol{1}_{\boldsymbol{n}_{\boldsymbol{i}}}$, where $\Delta\mathbb{\in R}$ and $\boldsymbol{1}_{\boldsymbol{n}_{\boldsymbol{i}}}={(1,1,\cdots,1)}^{T}$.

**Proof:** Since each row of $\boldsymbol{X}^{\left[ \boldsymbol{i} \right]}$ has only two non-zero elements -1 and 1, $\boldsymbol{b}={(\Delta,\Delta,\cdots,\Delta)}^{T}=\Delta\cdot\boldsymbol{1}_{\boldsymbol{n}_{\boldsymbol{i}}}$ are solutions. On the other hand, if one component of $\boldsymbol{b}$ is definite, such as $b_{1}=\Delta$, then all the components of $\boldsymbol{b}$ equal to $\Delta$, since the sub-model of $\boldsymbol{X}^{\left[ \boldsymbol{i} \right]}$ corresponds to a weakly connected component, so that any $b_{j}$ of $\boldsymbol{b}$ can be connected to $b_{1}$ by a path in the underlying undirected graph. Each edge in the path corresponds to an equation $b_{l}-b_{k}=0$, so that we have $b_{j}=b_{1}=\Delta$. Therefore, $\boldsymbol{b}= \Delta\cdot\boldsymbol{1}_{\boldsymbol{n}_{\boldsymbol{i}}}$ are all the solutions to the linear equations system $\boldsymbol{X}^{\left[ \boldsymbol{i} \right]}\boldsymbol{b}=\boldsymbol{0}$.

**Remark:** Thus, the dimension of the solution space to the linear equations system $\boldsymbol{X}^{\left[ \boldsymbol{i} \right]}\boldsymbol{b}=\boldsymbol{0}$ is 1, which is equivalent to $\mathrm{rank}{\boldsymbol{(}\boldsymbol{X}}^{\left[ \boldsymbol{i} \right]}{)=n}_{i}-1$. If $\boldsymbol{\beta}^{[\boldsymbol{i}]}$ is a set of parameters to the sub-model $\boldsymbol{Y}^{\left[ \boldsymbol{i} \right]}=\boldsymbol{X}^{\left[ \boldsymbol{i} \right]}\boldsymbol{\beta}^{\left[ \boldsymbol{i} \right]}+\boldsymbol{\varepsilon}^{\left[ \boldsymbol{i} \right]}$, then all the parameters satisfying the sub-model are $(\boldsymbol{\beta}^{[\boldsymbol{i}]}+\Delta\cdot\boldsymbol{1}_{\boldsymbol{n}_{\boldsymbol{i}}})$, since $\boldsymbol{X}^{\left[ \boldsymbol{i} \right]}{\tilde{\boldsymbol{\beta}}}^{\left[ \boldsymbol{i} \right]}=\boldsymbol{X}^{\left[ \boldsymbol{i} \right]}\boldsymbol{\beta}^{\left[ \boldsymbol{i} \right]}\Leftrightarrow\boldsymbol{X}^{\left[ \boldsymbol{i} \right]}\left( {\tilde{\boldsymbol{\beta}}}^{\left[ \boldsymbol{i} \right]}-\boldsymbol{\beta}^{\left[ \boldsymbol{i} \right]} \right)=\boldsymbol{0}\Leftrightarrow{\tilde{\boldsymbol{\beta}}}^{\left[ \boldsymbol{i} \right]}-\boldsymbol{\beta}^{\left[ \boldsymbol{i} \right]}=\Delta\cdot\boldsymbol{1}_{\boldsymbol{n}_{\boldsymbol{i}}}\Leftrightarrow{\tilde{\boldsymbol{\beta}}}^{\left[ \boldsymbol{i} \right]}=\boldsymbol{\beta}^{[\boldsymbol{i}]}+\Delta\cdot\boldsymbol{1}_{\boldsymbol{n}_{\boldsymbol{i}}}$. These parameters correspond to the same layout since each read translates the same distance. If we add an initial position of one read, such as $\beta_{j_{1}^{(i)}}=0$, to each sub-model $\boldsymbol{Y}^{\left[ \boldsymbol{i} \right]}=\boldsymbol{X}^{\left[ \boldsymbol{i} \right]}\boldsymbol{\beta}^{\left[ \boldsymbol{i} \right]}+\boldsymbol{\varepsilon}^{\left[ \boldsymbol{i} \right]}$, then to the design matrix $\boldsymbol{X}^{\left[ \boldsymbol{i} \right]}$ is added a row $\left( 1,0,\cdots,0 \right)$ with only the first component being non-zero, so that the solution to the linear equations system $\boldsymbol{X}^{\left[ \boldsymbol{i} \right]}\boldsymbol{b}=\boldsymbol{0}$ is exactly $\boldsymbol{b}=\boldsymbol{0}$. That is, the rank of $\boldsymbol{X}^{\left[ \boldsymbol{i} \right]}$ becomes $n_{i}$, i.e., full-rank in column. Hence, the regression parameters in each sub-model become unique, i.e., identifiable. Since $\mathrm{rank}\left( \boldsymbol{X} \right)\boldsymbol{=}\sum_{i=1}^{s} {rank(\boldsymbol{X}}^{\left[ \boldsymbol{i} \right]})=\sum_{i=1}^{s} n_{i}=n$, $\boldsymbol{X}$ also becomes full-rank in column, so that the parameters in the initial model $\boldsymbol{Y}=\boldsymbol{X\beta}+\boldsymbol{\varepsilon}$ become identifiable.

We consider the two pseudo reads generated from the contig ends flanking the gap. Their coordinates are respectively 0 and $g+l$, where $g$ is the gap size and $l$ is the read length. If either of them is contained in the contig, its coordinate could be used as the initial position added to the sub-model. Otherwise, the prior position of an arbitrary read could be used. In *de novo* assembly, where the prior positions of reads are unavailable, we could assign an arbitrary read a zero coordinate as the initial position. The choice of the initial position will not alter the layout of reads.

## Note S5. Sparsity of the coefficient matrix $\boldsymbol{X}^{\boldsymbol{T}}\boldsymbol{W}^{\left( \boldsymbol{k} \right)}\boldsymbol{X}$

**Proposition:** The sparsity (proportion of zeros) of $\boldsymbol{X}^{T}\boldsymbol{W}^{\left( k \right)}\boldsymbol{X}$ equals to $1-\frac{n+2m}{n^{2}}$, where $\boldsymbol{X}\in\mathbb{R}^{m\times n}$ is the design matrix and $\boldsymbol{W}^{\left( k \right)}$ is the weight matrix.

**Proof:** According to the regression model representing reads and overlaps, we observe that the design matrix $\boldsymbol{X}$ (illustrated in Fig. 1c) is actually the oriented incidence matrix of the overlap graph. So that $\boldsymbol{X}^{T}\boldsymbol{W}^{\left( k \right)}\boldsymbol{X}$ resembles a weighted version of the Laplacian matrix of the overlap graph, and non-zero elements exist only among the diagonal elements and the adjacency matrix elements. Specifically, the $r$^th^ diagonal element of $\boldsymbol{X}^{T}\boldsymbol{W}^{\left( k \right)}\boldsymbol{X}$ is the weighted degree of the $r$^th^ read in the overlap graph, namely, the sum of the weights for all the observations related with the $r$^th^ read. For the off-diagonal elements, only when the $i$^th^ read overlaps with the $j$^th^ read can the $(i,j)$^th^ element of $\boldsymbol{X}^{T}\boldsymbol{W}^{\left( k \right)}\boldsymbol{X}$ be a non-zero value, which equals to the negative weight assigned to that observation.

Therefore, the total number of non-zero elements in $\boldsymbol{X}^{T}\boldsymbol{W}^{\left( k \right)}\boldsymbol{X}\in\mathbb{R}^{n\times n}$ is $n+2m$, where $n$ means the number of diagonal elements and $m$ denotes the total number of overlapping read pairs; since $\boldsymbol{X}^{T}\boldsymbol{W}^{\left( k \right)}\boldsymbol{X}$ is symmetric, each pair of overlapping reads is counted twice. Therefore, we have that the sparsity of $\boldsymbol{X}^{T}\boldsymbol{W}^{\left( k \right)}\boldsymbol{X}$ is at least

$$1-\frac{n+2m}{n^{2}}.$$

**Remark:** $1-\frac{n+2m}{n^{2}}$ can be reformulated as $1-\frac{1+2m/n}{n}$, where $\frac{2m}{n}$ means the average number of overlaps of each read. So intuitively, $\frac{2m}{n}\ll n$ when the target sequence length is large, so that the coefficient matrix $\boldsymbol{X}^{T}\boldsymbol{W}^{\left( k \right)}\boldsymbol{X}$ is highly sparse. In the tandem repeat example of the *S. aureus* dataset, the design matrix had a dimension $5738\times530$, so the sparsity of $\boldsymbol{X}^{T}\boldsymbol{W}^{\left( k \right)}\boldsymbol{X}$ was 95.73%. It is extremely sparse, so we can take advantage of the efficient manipulation of sparse matrix.

## Note S6. Bayesian posterior probability per nucleotide type at each base site

In the gap sequence determination stage, after generating the multiple sequence alignment,

we calculate the Bayesian posterior probability of each nucleotide type, given the nucleotides aligned to the base site along with their Phred quality scores [[4](#_ENREF_4)]. We denote the base at the reference site as $S$, the aligned nucleotides as $\left\{ X_{i}=x_{i}, 1\leq i\leq m \right\}$ and their Phred scores as $\left\{ q_{i}, 1\leq i\leq m \right\}$. $S$ and ${\{X}_{i}\}$ take values from the alphabet $\mathcal{B=\{}A,G,C,T,-\}$. Here, the prior distribution of $S$ is assigned as a non-informative one, i.e., $\Pr\left( S=a \right)=1/5$.

According to the definition of the Phred quality score [[5](#_ENREF_5), [6](#_ENREF_6)], the scores can be interpreted as probabilities as follows,

$q_{i}=-10{log}_{10}(\varepsilon_{i})$,

where $\varepsilon_{i}$ is the error probability of base-calling, and $\varepsilon_{i}=\Pr\left( S\neq a \right| X_{i}=a)$. With the assumption of unbiased base-calling [[4](#_ENREF_4)], namely, $\Pr\left( X_{i}=a \right)=Pr(S=a)$, we have that

$\Pr\left( X_{i}=a | S=a \right)=\Pr\left( S=a \right| X_{i}=a)=1-\varepsilon_{i}$.

So that we assign the conditional probability as follows,

$$\Pr\left( X_{i}=x_{i} \right| S=a;q_{i})=\left\{ \begin{matrix} (1-\varepsilon_{i})(1-r), a\neq-,x_{i}=a \\ \begin{matrix} \frac{\varepsilon_{i}}{3}\left( 1-r \right), a\neq-,x_{i}\neq a,x_{i}\neq- \\ \begin{matrix} \\ \begin{matrix} r, a\neq-,x_{i}=- \\ \begin{matrix} 1-r, a=-,x_{i}=- \\ \frac{r}{4}, a=-,x_{i}\neq- \end{matrix} \end{matrix} \end{matrix} \end{matrix} \end{matrix} \right.$$

where $r$ is the indel error rate of the sequencing reads. Here, we assign $r=0.00005$.

With the above information, the posterior probability of $S$ being certain nucleotide type $a\in\{A,G,C,T,-\}$ is given by

$$\Pr\left( S=a | \left\{ X_{i}=x_{i} \right\};\left\{ q_{i} \right\} \right)=\frac{\Pr\left( \left\{ X_{i}=x_{i} \right\} \right| S=a; \left\{ q_{i} \right\})\cdot Pr(S=a)}{Pr( \left\{ X_{i}=x_{i} \right\}; \left\{ q_{i} \right\})}$$

$$=\frac{\prod_{i=1}^{m} \Pr\left( X_{i}=x_{i} \right| S=a; q_{i})\cdot Pr(S=a)}{\sum_{b\in\{A,G,C,T,-\}} (\prod_{i=1}^{m} \Pr\left( X_{i}=x_{i} \right| S=b; q_{i})\cdot\Pr\left( S=b \right))}$$

## Note S7. Command lines used in the Results section

| ***S. aureus* genome** | |
| --- | --- |
| GapCloser | GapCloser -a draft_genome.fasta -b config -o output_genome.fasta -l 101 -t 48 |
| GapFiller | perl GapFiller.pl -l libraries.txt -s draft_genome.fasta -b output -T 48 |
| Sealer | abyss-sealer -b20G -k90 -k80 -k70 -k60 -k50 -k40 -k30 -S ../draft_genome.fasta -o output 415_1.fq 415_2.fq -j 48 |
| Phrap | python RunPipeline_phrap.py -p prerequisite -g draft_genome.fasta -d output -t 48 |
| RegCloser | python RunPipeline.py -p prerequisite -g draft_genome.fasta -d output -t 48 |
| ***E. coli* genome** | |
| GapCloser | GapCloser -a draft_genome.fasta -b config -o output_genome.fasta -l 100 -t 48 |
| GapFiller | perl GapFiller.pl -l libraries.txt -s draft_genome.fasta -b output -i 20 -T 48 |
| Sealer | abyss-sealer -b20G -k90 -k80 -k70 -k60 -k50 -k40 -k30 -S draft_genome.fasta -o output1 lib3001.fq lib3002.fq lib5001.fq lib5002.fq lib8001.fq lib8002.fq rc_lib2k1.fq rc_lib2k2.fq rc_lib5k1.fq rc_lib5k2.fq -j 48  abyss-sealer -b20G -k90 -k80 -k70 -k60 -k50 -k40 -k30 -S output1_scaffold.fa -o output2 lib3001.fq lib3002.fq lib5001.fq lib5002.fq lib8001.fq lib8002.fq rc_lib2k1.fq rc_lib2k2.fq rc_lib5k1.fq rc_lib5k2.fq -j 48 |
| Phrap | python RunPipeline_phrap.py -p prerequisite -g draft_genome.fasta -d iter-1 -t 48  python RunPipeline_phrap.py -p prerequisite -g iter-1/output_genome.fasta -d iter-2 -t 48 |
| RegCloser | python RunPipeline.py -p prerequisite -g draft_genome.fasta -d iter-1 -w -t 48  python RunPipeline.py -p prerequisite -g iter-1/output_genome.fasta -d iter-2 -w -t 48 |
| **Plateau zokor genome** | |
| GapCloser | GapCloser -a draft_genome.fasta -b config1 -o output_genome1.fasta -l 150 -t 40  GapCloser -a output_genome1.fasta -b config2 -o output_genome2.fasta -l 150 -t 40 |
| GapFiller | perl GapFiller.pl -l libraries1.txt -s draft_genome.fasta -b output1 -i 1 -T 40  perl GapFiller.pl -l libraries2.txt -s output1/output1.gapfilled.final.fa -b output2 -i 1 -T 40 |
| Sealer | abyss-sealer -b20G -k90 -k80 -k70 -k60 -k50 -k40 -k30 -S draft_genome.fasta -o output1 300_R1.fq 300_R2.fq 500_R1.fq 500_R2.fq 800_R1.fq 800_R2.fq -j 40  abyss-sealer -b20G -k90 -k80 -k70 -k60 -k50 -k40 -k30 -S output1_scaffold.fa -o output2 300_R1.fq 300_R2.fq 500_R1.fq 500_R2.fq 800_R1.fq 800_R2.fq 3000_R1.fq 3000_R2.fq -j 40 |
| Phrap | python RunPipeline_phrap.py -p prerequisite1 -g draft_genome.fasta -d iter-1 -HO 10 -t 40  python RunPipeline_phrap.py -p prerequisite2 -g iter-1/output_genome.fasta -d iter-2 -rc 50 -HO 10 -mT 3000 -t 40 |
| RegCloser | python RunPipeline.py -p prerequisite1 -g draft_genome.fasta -d iter-1 -ho 5 -HO 10 -t 40  python RunPipeline.py -p prerequisite2 -g iter-1/output_genome.fasta -d iter-2 -ho 5 -HO 10 -mT 3000 -w -t 40 |
| RegCloser  (re-scaffolding) | python RunPipeline.py -p prerequisite1 -g draft_genome.fasta -d iter-1 -ho 5 -HO 10 -hf -rs -t 40  python RunPipeline.py -p prerequisite2 -g iter-1/output_genome.fasta -d iter-2 -ho 5 -HO 10 -hf -rs -t 40  python RunPipeline.py -p prerequisite3 -g iter-2/output_genome.fasta -d iter-3 -ho 5 -HO 10 -mT 3000 -w -hf -rs -t 40  python RunPipeline.py -p prerequisite4 -g iter-3/output_genome.fasta -d iter-4 -ho 5 -HO 10 -mT 3000 -w -hf -rs -t 40  python RunPipeline.py -p prerequisite5 -g iter-4/output_genome.fasta -d iter-5 -ho 5 -HO 10 -mT 3000 -w -hf -rs -t 40  python RunPipeline.py -p prerequisite6 -g iter-5/output_genome.fasta -d iter-6 -ho 5 -HO 10 -mT 3000 -w -hf -rs -t 40  python RunPipeline.py -p prerequisite7 -g iter-6/output_genome.fasta -d iter-7 -ho 5 -HO 10 -mT 3000 -w -hf -rs -t 40 |

**Computing equipments:** Gap closing of the *S. aureus* genome and the *E. coli* genome were run on a 48-core server with Intel Xeon CPU E5-2697 v2 @ 2.70 GHz and 378 GB RAM. Gap closing of the plateau zokor genome was run on a 40-core server with Intel Xeon Silver 4114 CPU @ 2.20 GHz and 692 GB RAM.

## Note S8. The orientating algorithm used in the layout generation of TGS long reads

The orientating procedure adopts the similar strategy as that in RegScaf [[7](#_ENREF_7)], which takes a heuristic algorithm to minimize the Hamilton loss function. But the definitions of $a_{ij}$ and $b_{ij}$ are a little different. Here $a_{ij}$ denotes the sum of alignment scores of the overlaps which indicate inconsistent orientations between read $i$ and $j$, and $b_{ij}$ denotes the sum of alignment scores of those inconsistent-orientation overlaps. When constructing the orientation graph, the edge weight is set to $p_{ij}=max(a_{ij},b_{ij})$, and the edge flag is set to the orientation signal ($if a_{ij}>b_{ij}then D_{ij}=1 ,else D_{ij}=-1$).

The orientating algorithm first finds the maximum spanning tree, MST, of the orientation graph. Then it traverses the MST in a depth-first order. In depth-first searching, neighbors are visited in a weight-decreasing order. When visiting a new vertex, its orientation is determined by the orientation of its precursor and their edge flag. The pseudo-code is as follows:

# Supplementary Tables

## Table S1. Comparison of the five methods on the *S. aureus* sequencing dataset

|  | **Draft** | **GapCloser** | **GapFiller** | **Sealer** | **Phrap** | **RegCloser** |
| --- | --- | --- | --- | --- | --- | --- |
| Contig length | 2,861,725 | 2,964,018 | 2,942,355 | - | 2,933,012 | 2,957,268 |
| Contig number | 703 | 169 | 228 | - | 188 | 200 |
| Contig N50 | 14,913 | 200,474 | 88,933 | - | 175,593 | **214,205** |
| Genome fraction | 95.633% | **98.927%** | 98.712% | - | 98.589% | 98.917% |
| # mis-assemblies | 1 | 2 | **1** | - | **1** | **1** |
| # local mis-assemblies | 0 | **3** | 4 | - | 5 | **3** |
| # mismatches | 14 | 315 | **52** | - | 194 | 108 |
| # indels | 6 | 37 | **23** | - | 45 | **23** |

Notes: Sealer did not finish after running for more than 72 hours.The assemblies were evaluated by aligning to the reference genome using QUAST (version 5.2.0) [[8](#_ENREF_8)]. The best values of each quality metric are highlighted in bold. RegCloser achieves the largest contig N50 with the fewest mis-assemblies and local mis-assemblies. Its genome fraction is 98.917%, close to the best value 98.927%.

## Table S2. Detailed information of the 6 simulation libraries of *E. coli*

| **Library number** | **PE or MP** | **Mean of**  **insert size (bp)** | **SD of**  **insert size (bp)** | **Coverage** | **Read length (bp)** |
| --- | --- | --- | --- | --- | --- |
| 1 | PE | 300 | 20 | 20X | 100 |
| 2 | PE | 500 | 30 | 20X | 100 |
| 3 | PE | 800 | 50 | 20X | 100 |
| 4 | MP | 2k | 200 | 20X | 100 |
| 5 | MP | 5k | 300 | 20X | 100 |
| 6 | MP | 10k | 500 | 20X | 100 |

Notes: PE, paired-end; MP, mate-pair; SD, standard deviation.

## Table S3. Detailed information of the 26 TR-related gaps on the *E. coli* draft genome and their closure results from the five methods

| **Gap No.** | **PS** | **CN** | **PM** | **PI** | **CN in gap** | **GapCloser** | **GapFiller** | **Sealer** | **Phrap** | **RegCloser** |
| --- | --- | --- | --- | --- | --- | --- | --- | --- | --- | --- |
| 80 | 113 | 5.9 | 96 | 0 | 4.9 |  |  |  |  | ✓ |
| 129 | 113 | 5.3 | 97 | 0 | 4.3 |  |  |  |  | ✓ |
| 130 | 100 | 5.8 | 96 | 0 | 4.3 |  |  |  |  | ✓ |
| 43 | 181 | 3.4 | 99 | 0 | 3.1 |  |  |  |  | ✓ |
| 120 | 92 | 4.4 | 95 | 0 | 3.1 |  |  |  |  | ✓ |
| 47 | 178 | 3.1 | 95 | 0 | 2.8 | ✓ |  |  |  | ✓ |
| 16 | 93 | 3.9 | 96 | 0 | 2.7 |  |  | ✓ |  | ✓ |
| 19 | 101 | 4.4 | 95 | 0 | 2.7 |  |  | ✓ | ✓ | ✓ |
| 46 | 535 | 2.9 | 97 | 0 | 2.5 |  |  |  | ✓ | ✓ |
| 97 | 113 | 3.5 | 96 | 0 | 2.4 | ✓ |  | ✓ |  | ✓ |
| 91 | 110 | 2.7 | 98 | 1 | 2.2 |  |  |  |  | ✓ |
| 135 | 121 | 2.7 | 89 | 2 | 2.2 | ✓ |  | ✓ | ✓ | ✓ |
| 85 | 122 | 2.7 | 87 | 2 | 2.1 | ✓ |  |  |  | ✓ |
| 132 | 111 | 2.7 | 98 | 0 | 2.1 |  |  |  |  | ✓ |
| 37 | 304 | 1.9 | 97 | 0 | 1.9 | ✓ |  |  |  | ✓ |
| 86 | 91 | 3.0 | 97 | 0 | 1.9 |  |  |  |  | ✓ |
| 17 | 93 | 4.1 | 97 | 0 | 1.6 |  |  |  |  | ✓ |
| 36 | 380 | 2.0 | 94 | 0 | 1.6 | ✓ |  |  |  | ✓ |
| 53 | 294 | 3.2 | 90 | 0 | 1.5 |  | ✓ |  |  | ✓ |
| 59 | 178 | 2.2 | 95 | 0 | 1.5 |  | ✓ |  | ✓ | ✓ |
| 115 | 98 | 2.9 | 92 | 1 | 1.5 |  |  |  |  | ✓ |
| 99 | 91 | 3.5 | 96 | 0 | 1.4 |  |  |  | ✓ | ✓ |
| 121 | 123 | 2.8 | 97 | 0 | 1.3 | ✓ |  |  |  | ✓ |
| 3 | 85 | 3.1 | 96 | 0 | 1.2 |  |  | ✓ | ✓ | ✓ |
| 18 | 200 | 2.0 | 96 | 0 | 1.2 |  | ✓ |  |  | ✓ |
| 136 | 101 | 4.1 | 95 | 0 | 1.0 | ✓ | ✓ | ✓ |  | ✓ |

Notes: PS, period size of the tandem repeat; CN, copy number of the tandem repeat; PM, percent of matches between adjacent copies overall; PI, percent of indels between adjacent copies overall; CN in gap, copy number of the tandem repeat contained in the gap region. The tick means the method correctly closes the corresponding gap. RegCloser correctly closes all the 26 TR-related gaps.

The gap records are sorted by CN in gap, and are partitioned into three sections with CN in gap respectively $>3$, $\leq3$ but $>1.5$, and $\leq1.5$. Intuitively, the gaps containing more repeat copies are more difficult to be correctly closed. When CN in gap $>1.5$, GapFiller fails to resolve any one gap. When CN in gap $>3$, none of the other four methods correctly closes any one gap.

## Table S4. Detailed information of the 7 sequencing libraries of the plateau zokor genome

| **Library number** | **PE or MP** | **insert size (bp)** | **SD of**  **insert size (bp)** | **Coverage (X)** | **Read length (bp)** |
| --- | --- | --- | --- | --- | --- |
| 1 | PE | 300 | 36 | 14.6 | 100 |
| 2 | PE | 500 | 42 | 15.0 | 100 |
| 3 | PE | 800 | 55 | 13.4 | 100 |
| 4 | MP | 3k | 335 | 27.7 | 100 |
| 5 | MP | 5k | 520 | 5.6 | 100 |
| 6 | MP | 8k | 560 | 12.5 | 100 |
| 7 | MP | 10k | 761 | 6.3 | 100 |

Notes: PE, paired-end; MP, mate-pair; SD, standard deviation.

## Table S5. Runtime and memory usage of the five gap-closing tools on the three data sets

| ***S. aureus*** | | | | | |
| --- | --- | --- | --- | --- | --- |
|  | **GapCloser** | **GapFiller** | **Sealer** | **Phrap** | **RegCloser** |
| Wall time (min) | 0.1 | 0.9 | - | 1.6 | 9.7 |
| Peak memory (Gb) | 0.4 | 0.2 | - | 1.3 | 7.7 |
| ***E. coli*** | | | | | |
| **Iteration 1** | **GapCloser** | **GapFiller** | **Sealer** | **Phrap** | **RegCloser** |
| Wall time (min) | 0.5 | 0.8 | 6.0 | 2.7 | 13.2 |
| Peak memory (Gb) | 0.7 | 0.6 | 20.1 | 1.6 | 7.9 |
| **Iteration 2** | **GapCloser** | **GapFiller** | **Sealer** | **Phrap** | **RegCloser** |
| Wall time (min) | - | 0.5 | 6.0 | 0.8 | 1.2 |
| Peak memory (Gb) | - | 0.7 | 20.1 | 0.4 | 0.3 |
| **Total** | **GapCloser** | **GapFiller** | **Sealer** | **Phrap** | **RegCloser** |
| Wall time (min) | 0.5 | 3.2 | 12.0 | 3.5 | 14.4 |
| Peak memory (Gb) | 0.7 | 0.7 | 20.1 | 1.6 | 7.9 |
| **Plateau zokor** | | | | | |
| **The first round** | **GapCloser** | **GapFiller** | **Sealer** | **Phrap** | **RegCloser** |
| Wall time (h) | 31.3 | 19.4 | 12.8 | 23.0 | 23.4 |
| Peak memory (Gb) | 550.0 | 9.8 | 21.6 | 37.4 | 237.4 |
| **The second round** | **GapCloser** | **GapFiller** | **Sealer** | **Phrap** | **RegCloser** |
| Wall time (h) | 58.1 | 36.2 | 18.5 | 45.9 | 52.8 |
| Peak memory (Gb) | 539.2 | 18.0 | 22.1 | 64.0 | 582.5 |

Notes: The command lines and computing equipments for running the five gap-closing tools on the three data sets are provided in Note S7. On the *S. aureus* data set, Sealer did not finish after running for more than 72 hours. On the *E. coli* data set, the five tools were run iteratively until no more gaps could be closed. GapCloser ran for 1 iteration; GapFiller ran for 7 iterations; Sealer, Phrap, and RegCloser all ran for 2 iterations. On the plateau zokor data set, the five tools were run by two rounds. In the first round, three paired-end libraries with insert sizes of 300, 500, and 800 bp were used. In the second round, a mate-pair library with a long insert size of 3000 bp was added.

**References**

1. Schirmer M, D'Amore R, Ijaz UZ, Hall N, Quince C: **Illumina error profiles: resolving fine-scale variation in metagenomic sequencing data**. *BMC Bioinformatics* 2016, **17**:125.

2. Schirmer M, Ijaz UZ, D'Amore R, Hall N, Sloan WT, Quince C: **Insight into biases and sequencing errors for amplicon sequencing with the Illumina MiSeq platform**. *Nucleic Acids Res* 2015, **43**(6):e37.

3. Arumugam S, Brandstädt A, Nishizeki T: **Handbook of graph theory, combinatorial optimization, and algorithms**, vol. 34: CRC Press; 2016.

4. Li M, Nordborg M, Li LM: **Adjust quality scores from alignment and improve sequencing accuracy**. *Nucleic Acids Res* 2004, **32**(17):5183-5191.

5. Ewing B, Green P: **Base-calling of automated sequencer traces using phred. II. Error probabilities**. *Genome Res* 1998, **8**(3):186-194.

6. Ewing B, Hillier L, Wendl MC, Green P: **Base-calling of automated sequencer traces using phred. I. Accuracy assessment**. *Genome Res* 1998, **8**(3):175-185.

7. Li M, Li LM: **RegScaf: a regression approach to scaffolding**. *Bioinformatics* 2022, **38**(10):2675-2682.

8. Gurevich A, Saveliev V, Vyahhi N, Tesler G: **QUAST: quality assessment tool for genome assemblies**. *Bioinformatics* 2013, **29**(8):1072-1075.
